# Nanoparticle-boosted myeloid-derived suppressor cell therapy for immune reprogramming in multiple sclerosis

**DOI:** 10.1126/sciadv.ady4135

**Published:** 2025-10-15

**Authors:** Endong Zhang, Hanan Algarni, Luyu Zhang, Chih-Jia Chao, Shan He, Aditi Upadhye, Qing Bao, Dahee Jung, Shubhi Srivastava, Edidiong Udofa, Philana Phan, Dejan S. Nikolic, Steve Seung-Young Lee, Jalees Rehman, Zongmin Zhao

**Affiliations:** ^1^ Department of Pharmaceutical Sciences, University of Illinois Chicago, Chicago, IL, USA.; ^2^ Department of Biochemistry and Molecular Genetics, College of Medicine, University of Illinois, Chicago, IL, USA.; ^3^ University of Illinois Cancer Center, Chicago, IL, USA.

## Abstract

Massive immune cell infiltration and persistent inflammation in the central nervous system (CNS) are key hallmarks of multiple sclerosis. Here, we report a myeloid-derived suppressor cell (MDSC)–based therapeutic strategy, named CNS Immune Targeting Enabled by MDSCs (CITED), which uses surface-decorated MDSCs carrying rapamycin nanoparticles (NPs) for targeted multimodal immune reprogramming in CNS. We show that NP decoration enhances MDSC immunomodulatory function, facilitates their trafficking to inflamed CNS regions, and increases NP accumulation within CNS. In an experimental autoimmune encephalomyelitis model, CITED exhibited robust therapeutic efficacy, resulting in reduced disease progression, improved motor function, and diminished myelin damage. Mechanistic studies reveal that CITED exerts its therapeutic effects by targeted reprogramming of both innate and adaptive immune responses in CNS. Specifically, CITED inhibits immune cell infiltration, rebalances CD4 T cell phenotypes, and promotes the polarization of myeloid cells toward anti-inflammatory phenotypes. Collectively, CITED could provide a broadly effective approach for targeted immune restoration in multiple sclerosis and potentially other autoimmune diseases.

## Teaser

Nanoparticle-armed MDSCs restore CNS immune homeostasis, unlocking a path for targeted MS and autoimmune therapies.

## INTRODUCTION

Multiple sclerosis (MS) is a chronic inflammatory autoimmune disease characterized by immune tolerance breakdown, persistent inflammation, and demyelination within the central nervous system (CNS) (*1*, *2*). These pathological changes result in progressive neurodegeneration and long-term disability. The exact causes of MS are complex and multifactorial, involving a combination of genetic and environmental triggers (e.g., virus infection, diet, and obesity) (*1*, *3*–*6*). Current treatments for MS primarily consist of disease-modifying therapies, including pan-immunosuppressive drugs, immune cell migration inhibitors, and immune cell–depleting agents (*7*–*10*). These therapies work by inhibiting or depleting CNS-homing, autoreactive immune cells to inhibit lesion development. However, while they can alleviate symptoms, they have only limited capacity to prevent or reverse disease progression. Moreover, most of these treatments rely on global immunosuppression and are not specific to immune cells in the CNS lesions due to their limited ability to cross the blood-brain barrier and inefficient delivery to the CNS (*7*, *11*). Such global immunosuppressive approaches increase the vulnerability of patients to infections by pathogens, thus highlighting the need for targeted therapies.

The disturbance of immune tolerance, leading to the activation and infiltration of autoreactive immune cells that attack the myelin sheath in the CNS, is a primary driver of MS progression (*1*, *2*). The onset and progression of MS involves a complex and dynamic interplay between innate and adaptive immune cells within the CNS. Accumulating evidence suggests that both the innate and adaptive immune cells create a vicious cycle that exacerbates disease progression (*1*, *2*, *7*, *11*, *12*). For instance, the massive infiltration of CD4 and CD8 T cells injures the myelin sheath (*13*, *14*). Moreover, the phenotypic shift of CD4 T cells toward proinflammatory subsets, such as T helper 1 (T_H_1) and T helper 17 (T_H_17) cells, coupled with a reduction in regulatory T (T_reg_) cells, further amplifies the autoimmune response (*12*, *15*–*19*). Beyond adaptive immunity, emerging studies reveal that innate immune cells, such as myeloid cells, also promote MS progression. For instance, infiltrating myeloid cells and resident microglia are frequently polarized to a proinflammatory phenotype in the CNS, thus perpetuating persistent neuroinflammation and tissue damage (*20*–*23*). Consequently, therapeutic strategies that could efficiently target the CNS and simultaneously reprogram both innate and adaptive immune responses to reestablish immune homeostasis are needed.

Here, we report a nanoengineered myeloid-derived suppressor cell (MDSC)–based approach, CNS Immune Targeting Enabled by MDSCs (CITED), as an effective therapeutic strategy for MS that enables targeted, multilevel immune reprogramming. MDSCs, a heterogeneous population of immature myeloid cells characterized by their immunosuppressive function (*24*–*26*), are critical immunomodulatory cells with context-dependent disease inhibiting or promoting roles in various autoimmune diseases including MS (*27*, *28*). While the precise mechanisms underlying MDSC function in MS remain incompletely understood, emerging evidence suggests that MDSCs accumulate in the CNS and peripheral tissues during MS, where they regulate both innate and adaptive immune responses (*28*). However, despite their multifaceted roles, the direct use of MDSCs as a cell therapy remains underexplored (*29*, *30*), and their therapeutic efficacy has been limited largely attributed to their high plasticity, suboptimal immunoregulatory function in dynamic disease immune environments, and rapid loss of suppressive activity due to cellular maturation and differentiation. The CITED strategy involves decorating MDSC surface with rapamycin-loaded polymeric nanoparticles (NPs) as “backpacks” to regulate their differentiation and enhance their immunomodulatory function. The CITED approach enables CNS-targeted, multimodal immune reprogramming aimed at restoring immune homeostasis in MS. By leveraging the inherent ability of MDSCs to actively home toward inflamed tissues (*24*, *31*), CITED facilitates efficient MDSC infiltration into the spinal cord and brain, resulting in enhanced rapamycin delivery. Moreover, stable anchoring of NPs to MDSC surface enables sustained rapamycin release, which autocrinally reinforces the immunosuppressive function of adoptively transferred MDSCs by modulating immunoregulatory signaling pathways such as nuclear factor κB (NF-κB) signaling (*32*). Together, CITED integrates functionally enhanced MDSCs with optimized rapamycin delivery to achieve robust, targeted modulation of both innate and adaptive immune responses. In an experimental autoimmune encephalomyelitis (EAE) model, we demonstrate that CITED induces multilevel immune reprogramming in the CNS, including suppression of immune cell infiltration, rebalancing CD4 T cell phenotypes, and polarization of myeloid cells toward anti-inflammatory phenotypes. These findings suggest that CITED could provide a promising and broadly effective strategy for targeted immunotherapy in MS and potentially other autoimmune disorders.

## RESULTS

### Design, fabrication, and characterization of CITED

**Fig. 1.**
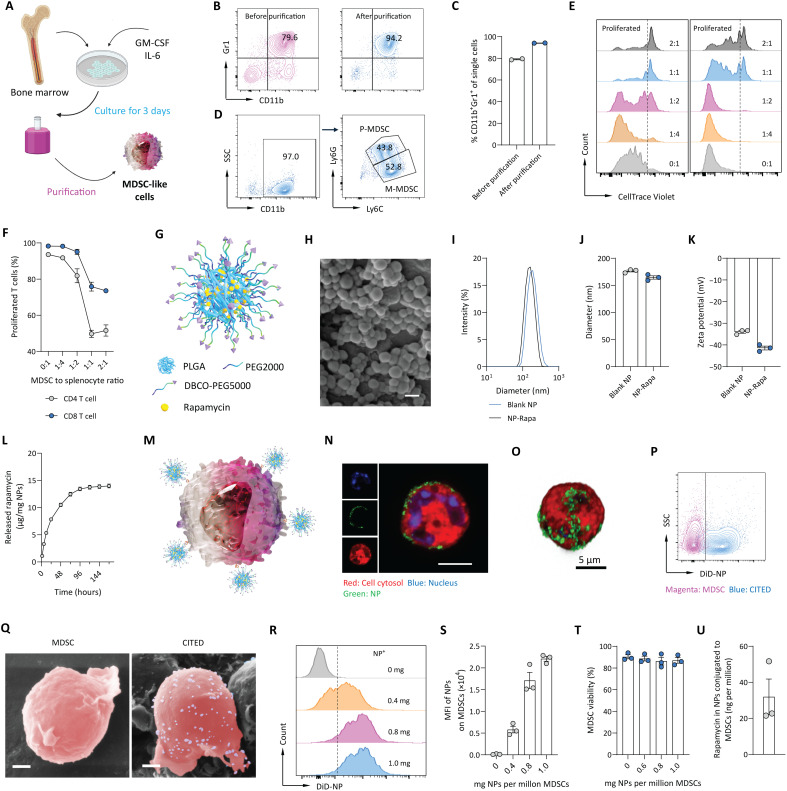
Design and characterization of immunomodulatory NP decorated MDSCs (CITED). (**A**) Schematic showing the method to culture MDSC-like cells. Created in BioRender: Z. Zhao (2025); https://BioRender.com/2tie67h. (**B** and **C**) Characterization of the expression of MDSC markers (CD11b and Gr1) on MDSC-like cells. Representative flow cytometry plots (B) and quantification of the purity of obtained MDSCs (C) are shown. (**D**) Representative flow cytometry plots showing that the obtained MDSCs contain both M-MDSCs and P-MDSCs. (**E** and **F**) Characterization of the immunosuppressive activity of MDSCs in inhibiting proliferation of activated T cells. (E) Representative flow cytometry plots of CellTrace Violet–stained CD4 or CD8 T cells after coculture with MDSCs in the presence of anti-CD3 and anti-CD28 antibodies for 5 days. (F) Percentage of proliferated CD4 or CD8 T cells following coculture with MDSCs for 5 days. (**G** to **L**) Design and characterization of rapamycin-loaded PLGA NPs (NP-Rapa).(G) Schematic showing the design of NP-Rapa NPs. (H) Representative SEM image of NP-Rapa NPs. Scale bar, 200 nm. (I to K) Size distribution (I), average diameter (J), and zeta potential (K) of NP-Rapa NPs. (L) Release profile of rapamycin from NP-Rapa NPs in PBS. (**M** to **U**) Design and characterization of decoration of NPs to MDSCs. (M) Schematic showing the design of CITED. [(N) and (O)] 2D (N) and reconstructed 3D (O) confocal fluorescence microscopy images of P-MDSCs decorated with NPs. (P) Flow cytometry of native MDSCs or MDSCs decorated with DiD-labeled NPs. (Q) Pseudocolored SEM images of a P-MDSC carrying NPs. P-MDSC and NPs were pseudocolored in pink and purple, respectively. Scale bars, 1 μm. [(R) and (S)] Representative flow cytometry plot (R) and corresponding quantification (S) of the number of NPs on MDSCs as indicated by mean fluorescence intensity (MFI) at different NP feed conditions. (T) Viability of MDSCs conjugated with NPs. (U) Amount of rapamycin carried by MDSCs in CITED. Data in (C), (F), (J) to (L), and (S) to (U) are presented as means ± SEM.

We first developed a robust method to derive MDSCs from bone marrow cells (Fig. 1A). This method involves culturing bone marrow cells with two proinflammatory cytokines, granulocyte-macrophage colony-stimulating factor (GM-CSF) and interleukin-6 (IL-6), for 3 days (*33*), followed by a purification step. Using this method, we achieved a high purity of cells (>94%) (Fig. 1, B and C), which express the MDSC characteristic surface markers CD11b and Gr1 (*26*, *34*) (Fig. 1, B and C). Natural MDSCs include two subtypes: polymorphonuclear MDSCs (P-MDSCs; CD11b^+^Ly6G^+^Ly6C^lo^) and monocytic MDSCs (M-MDSCs; CD11b^+^Ly6G-Ly6C^high^) (*26*). Our method yielded a mixture of both subtypes (Fig. 1D), mimicking the heterogeneity of natural MDSCs. To functionally validate these MDSC-like cells, we performed a T cell suppression assay via coculturing MDSC-like cells and splenocytes to measure their suppression effect toward proliferation of activated T cells, a characteristic function of natural MDSCs (*26*, *34*). Our results demonstrated that the MDSC-like cells effectively inhibited the proliferation of both CD4 and CD8 T cells (activated by anti-CD3 and anti-CD28 antibodies) in a dose-dependent manner (Fig. 1, E and F, and fig. S1). For simplicity, we refer to these MDSC-like cells as MDSCs unless mentioned otherwise.

Next, we designed and synthesized a biodegradable immunomodulatory NP (NP-Rapa) composed of poly(lactide-*co*-glycolide) (PLGA), which encapsulates rapamycin and displays reactive dibenzocyclooctyne (DBCO) ligands on its surface (Fig. 1G). The resulting NP-Rapa NPs are spherical, with an average diameter of 176.4 nm, and have a negatively charged surface (Fig. 1, H to K). We measured the release kinetics of rapamycin from NP-Rapa NPs in phosphate-buffered saline (PBS) and found that the drug is released in a sustained manner over 7 days, with the majority being released within the first 72 hours (Fig. 1L). To decorate NP-Rapa NPs to the surface of MDSCs, we used a DBCO-azido click chemistry method (Fig. 1M) (*35*, *36*). This method involves introducing azido functional groups to the MDSC surface, allowing them to react with the DBCO ligands on NP-Rapa NPs. Our data confirmed that adding N-azidoacetylmannosamine-tetraacylated (Ac_4_ManNAz) during MDSC culture effectively introduced reactive azido groups to MDSC surface (fig. S2, A to C). MDSCs, without azido functionalization, did not efficiently internalize the NPs, likely due to their limited phagocytic activity (fig. S3, A to C). Using the click chemistry method, NPs were efficiently decorated onto the surface of both P-MDSCs and M-MDSCs, as demonstrated by our flow cytometry, confocal laser fluorescence microscopy (CLSM), and scanning electron microscopy (SEM) data (Fig. 1, N to Q, and fig. S4, A and B). We further optimized the conjugation process by adjusting the feed ratio of NPs to MDSCs. The decoration of NPs on MDSCs reached saturation when 1 mg of NPs per million MDSCs was used, without substantially affecting MDSC’s viability (Fig. 1, R to T). Under these conditions, each million MDSCs carried ~32 ng of rapamycin (Fig. 1U). In addition, rapamycin release from CITED in PBS containing 10% fetal bovine serum (FBS) exhibited an initial burst phase followed by a sustained release pattern (fig. S5). We used this lead CITED formulation for the following studies.

### Surface conjugation of NPs enhances the immunomodulatory activity of CITED

**Fig. 2.**
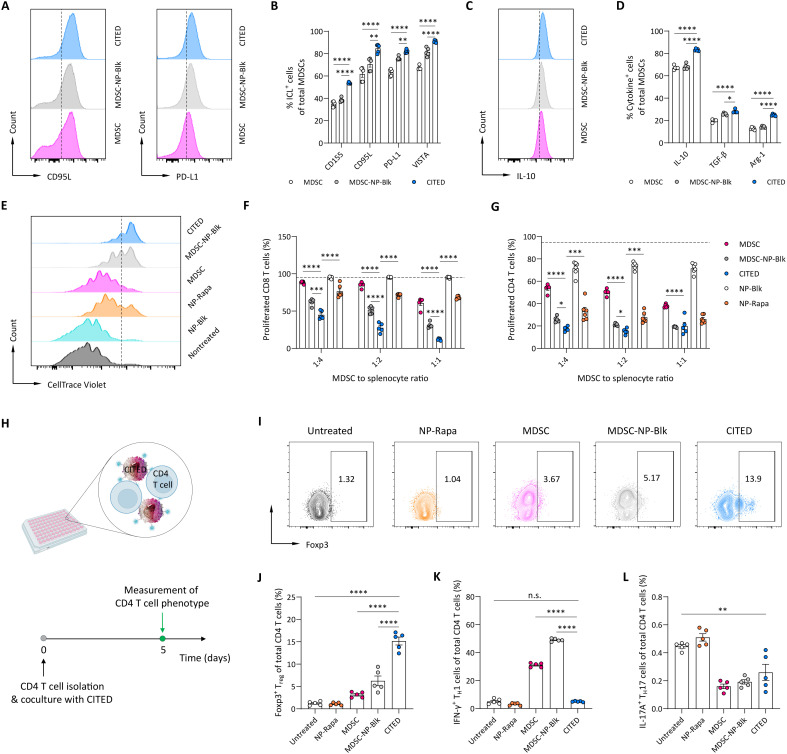
Cell-surface conjugation of rapamycin NPs enhances the immunomodulatory activity of CITED. (**A** to **D**) NP conjugation led to higher expression of immune checkpoint ligands and anti-inflammatory cytokines/factors in MDSCs. (A) Representative flow cytometry plot showing the expression of CD95L and PD-L1 on MDSCs. (B) Percentage of MDSCs expressing immune checkpoint ligands (ICLs). (C) Representative flow cytometry plot showing the expression of IL-10 in MDSCs following NP conjugation. (D) Percentage of MDSCs expressing anti-inflammatory factors. In (A) to (D), marker expression was measured 48 hours after NP conjugation. (**E** to **G**) NP conjugation enhanced MDSC’s capability to inhibit T cell proliferation. (E) Representative flow cytometry plot showing the proliferation of CD8 T cells after coculturing with different formulations for 5 days, at an MDSC to splenocyte ratio of 1:4 or equivalent amount of NPs, in the presence of anti-CD3 and anti-CD28 antibodies to activate T cells. (F) Percentage of proliferated CD8 T cells 5 days after coculture. (G) Percentage of proliferated CD4 T cells 5 days after coculture. In (F) to (G), the dotted lines indicate the average percentage of proliferated T cells in nontreated splenocytes. (**H** to **L**), CITED modulated the phenotype of CD4 T cells in a coculture setup. (H) Schematic showing the experimental design. Created in BioRender: Z. Zhao (2025); https://BioRender.com/2tie67h. (I) Representative flow cytometry plots showing the relative number of Foxp3^+^ T_reg_ cells. [(J) to (L)] The relative number of Foxp3^+^ T_reg_ cells (J), IFN-γ^+^ T_H_1 cells (K), and IL-17A^+^ T_H_17 cells (L) after coculturing CD4 T cells with CITED or control formulations in the presence of anti-CD3 and anti-CD28 antibodies for 5 days. Data in (B), (D), (F), (G), and (J) to (L) are presented as means ± SEM. For (B), (D), (F), (G), and (J) to (L), statistical analysis was conducted by one-way analysis of variance (ANOVA) with Dunnett’s test: *P < 0.05; **P < 0.01; ***P < 0.001; ****P < 0.0001. n.s., not significant.

CITED relies on surface-anchored NPs as “backpacks” to store and sustainably release rapamycin, which autocrinally regulates MDSC differentiation and enhances their immunomodulatory function. We conducted a set of experiments to determine the impact of NP conjugation on the immunomodulatory activity of CITED. First, we assessed the expression of immune checkpoint ligands and anti-inflammatory cytokines in MDSCs (Fig. 2, A to D), two key mechanisms underlining the immunosuppressive activity of MDSCs (*37*). Through cell contact–dependent mechanisms, such as immune checkpoint interactions, MDSCs can effectively suppress T cell proliferation (*37*). Via cell contact–independent pathways, MDSCs secrete anti-inflammatory cytokines that not only inhibit T cell proliferation but also influence the phenotype of T cells and innate immune cells, including macrophages and dendritic cells (DCs) (*25*, *38*). Compared to control MDSCs and MDSCs conjugated with blank PLGA NPs (MDSC-NP-Blk), CITED exhibited a significantly higher expression of immune checkpoint ligands, including CD155, CD95L, programmed death ligand-1 (PD-L1), and V-domain immunoglobulin suppressor of T cell activation (VISTA) (Fig. 2, A and B), showing a 1.3- to 1.5-fold increase over MDSCs alone. Notably, conjugation of blank PLGA NPs to MDSCs also significantly increased the expression of PD-L1 and VISTA. This effect is possibly attributed to lactic acid released from PLGA NP degradation, which has been reported to modulate myeloid cell metabolism and polarize them toward immunosuppressive phenotypes (*39*–*41*). Moreover, we also observed markedly increased expression of anti-inflammatory mediators such as IL-10, transforming growth factor–β (TGF-β), and arginase-1 on MDSCs when surface-decorated with NP-Rapa NPs (Fig. 2, C and D). For example, CITED led to a ~1.2-fold increase in IL-10–expressing cells compared to MDSCs alone or MDSCs conjugated with blank NPs (Fig. 2D). Our reverse transcription quantitative polymerase chain reaction (RT-qPCR) results validated the higher expression of PD-L1 and TGF-β of CITED at the mRNA level (fig. S6). The enhanced expression of immune checkpoint ligands and anti-inflammatory cytokines in CITED can contribute to increased immunosuppressive activity through cell contact–dependent and cell contact–independent mechanisms, respectively.

To functionally evaluate the immunomodulatory activity of CITED, we performed a T cell coculture assay to evaluate its inhibitory effect on proliferation of activated T cells. Across various MDSC to splenocyte ratios, CITED demonstrated a significantly stronger inhibitory effect on the proliferation of both CD8 and CD4 T cells (activated by anti-CD3 and anti-CD28 antibodies) compared to MDSCs alone or MDSCs conjugated with blank NPs (Fig. 2, E to G). Specifically, CITED showed a 2.0- to 5.0-fold greater inhibitory effect on CD8 T cell proliferation and a 1.9- to 3.3-fold increase in inhibition of CD4 T cell proliferation compared to MDSCs alone. Notably, MDSCs conjugated with blank PLGA NPs also showed markedly stronger inhibition of CD8 and CD4 T cell proliferation, likely due to the increased surface expression of PD-L1 and VISTA. However, their effect was not as potent as that of CITED, indicative of the synergistic effects of NP conjugation and use of rapamycin. Beyond directly inhibiting T cell proliferation, MDSCs can modulate CD4 T cell phenotypes and promote their transition to anti-inflammatory T_reg_ cells (*42*, *43*). To assess CITED’s capability to modulate CD4 T cell phenotypes, we performed a CD4 T cell–MDSC coculture assay and measured the relative abundance of T_reg_ cells, T_H_1 cells, and T_H_17 cells, 5 days post-coculture (Fig. 2H). Coculturing CD4 T cells with MDSCs alone significantly increased the percentage of both Foxp3-expressing T_reg_ cells and interferon-γ (IFN-γ)–expressing T_H_1 cells compared to untreated CD4 T cells (Fig. 2, I to K). Conjugation of NP-Rapa NPs to MDSCs (CITED) further enhanced their immunomodulatory effect on CD4 T cell phenotypes. Specifically, CITED led to a 4.8-fold increase in T_reg_ cells and an 83.8% decrease in T_H_1 cells compared to MDSCs alone. In addition, CITED resulted in a 58.3% reduction in IL-17A–expressing T_H_17 cells compared to untreated CD4 T cells, and its effect is similar to that of MDSCs alone (Fig. 2L). Moreover, beyond their improved immunomodulatory activity on T cells, our data in a DC-MDSC coculture study suggest that CITED significantly reduced the expression of costimulatory molecules on DCs including CD80 and CD40, when stimulated with lipopolysaccharide and a model antigen ovalbumin (fig. S7, A to D). This indicates that CITED promoted the DCs toward the tolerogenic phenotype, which is critical for inducing and maintaining immune tolerance (*44*). Collectively, our findings suggest that Rapa NP conjugation to MDSCs significantly enhances MDSCs’ immunomodulatory activity.

We next analyzed how CITED affected the transcriptome of MDSCs by performing unbiased transcriptomic analysis using bulk RNA sequencing (RNA-seq) of control MDSCs, MDSCs decorated with blank NP, as well as MDSCs decorated with Rapa NP (figs. S8 to S11). MDSCs conjugated with either blank NP or Rapa NP showed markedly distinct transcriptome profiles (figs. S8, A and B, and S10A). Further gene set enrichment analysis (fig. S8C) showed that compared to MDSCs, CITED up-regulated genes related to immune response regulation pathways, such as genes related to negative regulation of immune response (fig. S9A), suggesting that Rapa NP decoration enhances MDSC immunomodulatory function. In addition, CITED also resulted in up-regulation of genes related to cell chemotaxis and locomotion (fig. S9, B and C), suggesting an augmented migratory potential of CITED compared to MDSCs. Concomitantly, genes associated with cell cycle progression and nuclear division were down-regulated by CITED (fig. S9D), indicative of reduced potential for cell proliferation of MDSCs. We also compared how conjugation of blank NP modulated the MDSC transcriptome (figs. S10 and S11). Conjugation of blank NPs up-regulated genes related to cell chemotaxis, cellular response to lipids, and exocytosis while down-regulating genes related to cell cycle progression.

### CITED traffics to and enhances the delivery of NPs to the CNS

**Fig. 3.**
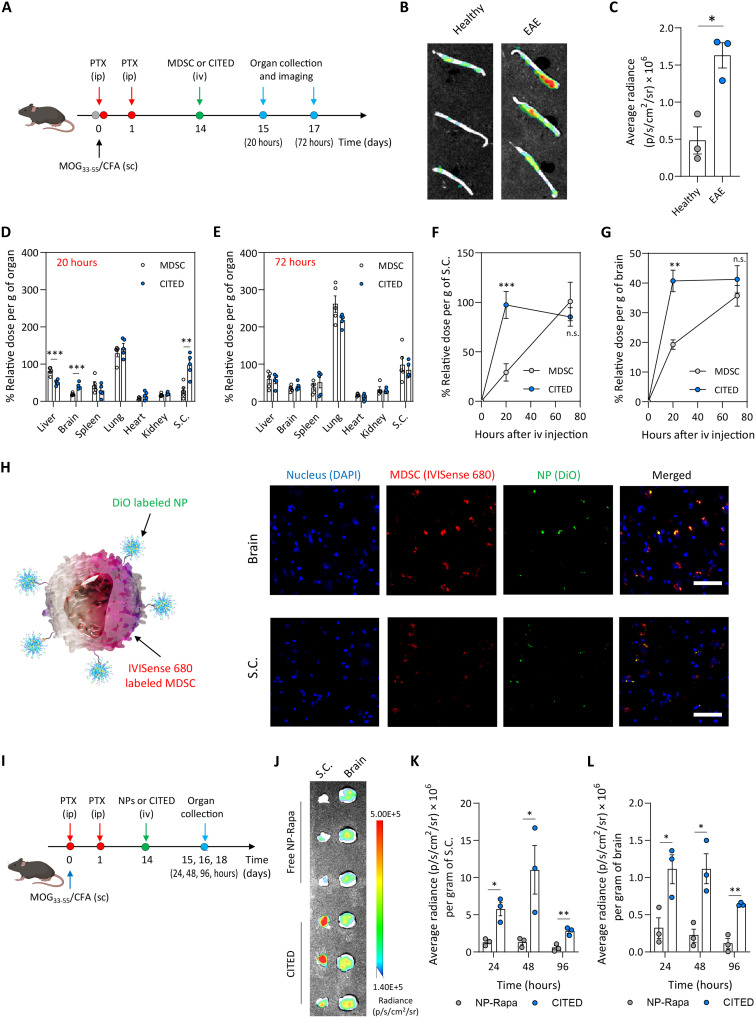
CITED effectively traffics to the CNS and enhances NP delivery to the CNS. (**A** to **G**) Biodistribution of CITED in an EAE model. (A) Schematic showing the schedule for the biodistribution study. Created in BioRender: Z. Zhao (2025); https://BioRender.com/2tie67h. (B) Representative Lago X images of the spinal cord (S.C.) of healthy or EAE mice 20 hours after intravenous (iv) injection of CITED (MDSCs labeled with IVISense 680). (C) Relative number of CITED MDSCs in the spinal cord of healthy versus EAE mice. [(D) and (E)] Biodistribution of MDSCs in major organs in the free form or in CITED, 20 hours (D) or 72 hours (E) after intravenous injection in the EAE model. [(F) and (G)] Comparison of the time-dependent MDSC accumulation in the spinal cord (F) or brain (G) after intravenous formulation administration. (**H**) Confocal fluorescence microscopy images showing the presence of CITED in the brain and spinal cord, 20 hours after intravenous injection. For CITED in this study, MDSCs and NPs were labeled by IVISense 680 and DiO, respectively. Scale bars, 50 μm. (**I** to **L**) CITED enhanced the delivery of NPs (NP-Rapa) to the CNS in an EAE model. (I) Schematic showing the schedule for the study. In this study, NP-Rapa NPs were fluorescently labeled by coloading DiR. Created with BioRender. (J) Lago X images showing the accumulation of NPs, either in the free or CITED form, to the brain and spinal cord 48 hours after intravenous injection. [(K) and (L)] Amount of NPs in the spinal cord (K) and brain (L). For (B) and (C), *n* = 3 biologically independent animals. For (D) to (G), *n* = 5 biologically independent animals. For (I) to (L), *n* = 3 biologically independent animals. Data in (C) to (G), (K), and (L) are presented as means ± SEM. For (C) to (G), (K), and (L), statistical analysis was conducted by two-tailed Student’s *t* test: *P < 0.05; **P < 0.01; ***P < 0.001. ip, intraperitoneal; sc, subcutaneous.

We next conducted a biodistribution study to evaluate the trafficking of CITED to the inflamed CNS in murine experimental autoimmune EAE (Fig. 3A). Previous studies have reported that chronic CNS inflammation and associated leaky blood-brain barrier are involved in the progression of EAE, a murine model of MS (*45*). As living myeloid cells, MDSCs express chemokine receptors and can actively migrate to the inflamed CNS via chemokine-chemokine receptor interactions (*31*). We observed that 20 hours after intravenous administration, CITED MDSCs exhibited a 3.4-fold higher accumulation in the spinal cord of EAE mice compared to healthy mice (Fig. 3, B and C), indicating their targeted trafficking capability toward the inflamed spinal cord.

Further biodistribution studies revealed that, in EAE mice, CITED showed significantly reduced accumulation in the liver and markedly higher accumulation in the brain and spinal cord compared to MDSCs alone at 20 hours postadministration (Fig. 3D). However, by 72 hours postinjection, CITED showed a similar biodistribution profile as MDSCs alone (Fig. 3E). Time-course analyses of cell accumulation in the CNS showed that CITED trafficked to the spinal cord and brain more rapidly than MDSCs alone (Fig. 3, F and G). This result is consistent with our RNA-seq data (fig. S8, C and E to F) showing CITED up-regulated genes related to cell chemotaxis and locomotion. Our data also suggested that NP conjugation to CITED resulted in up-regulated expression of chemokine receptors including CCR2 and CX3CR1, which could facilitate MDSC migration (fig. S12). In a separate study, we fluorescently colabeled MDSCs and NPs in CITED and assessed their trafficking to the spinal cord and brain through confocal fluorescence imaging of sectioned tissues. At 24 hours postinjection, we observed colocalized MDSCs and NPs in the brain and spinal cord of EAE mice (Fig. 3H), indicating that CITED migrated intact and efficiently carried surface-decorated NPs to the inflamed CNS.

To further evaluate NP delivery, we conducted a follow-up NP biodistribution study comparing the accumulation of free rapamycin-loaded NPs (NP-Rapa) versus CITED-delivered NPs in the CNS at various time points after intravenous administration (Fig. 3I). At all tested time points, compared with free NPs, CITED resulted in a 3.5- to 5.1-fold and 4.3- to 8.3-fold higher NP accumulation in the brain and spinal cord of EAE mice, respectively (Fig. 3, J to L, and fig. S13A). Consistently, rapamycin quantification in brain and spinal cord tissues showed that, while rapamycin was detectable only at 24 hours in mice treated with NP-Rapa, it was present at markedly higher levels across all three time points in the CITED group (fig. S13, B and C). Notably, NP and rapamycin accumulation in both the brain and spinal cord peaked at 48 hours and declined by 96 hours in CITED-treated mice (Fig. 3, K and L, and fig. S13, A to C). These findings suggest that CITED could enhance NP accumulation within the inflamed CNS. Collectively, our biodistribution results demonstrated that CITED could efficiently traffic to the injured CNS and enhance the accumulation of surface-decorated NPs to inflamed CNS lesions.

### CITED shows robust therapeutic efficacy in an EAE model

**Fig. 4.**
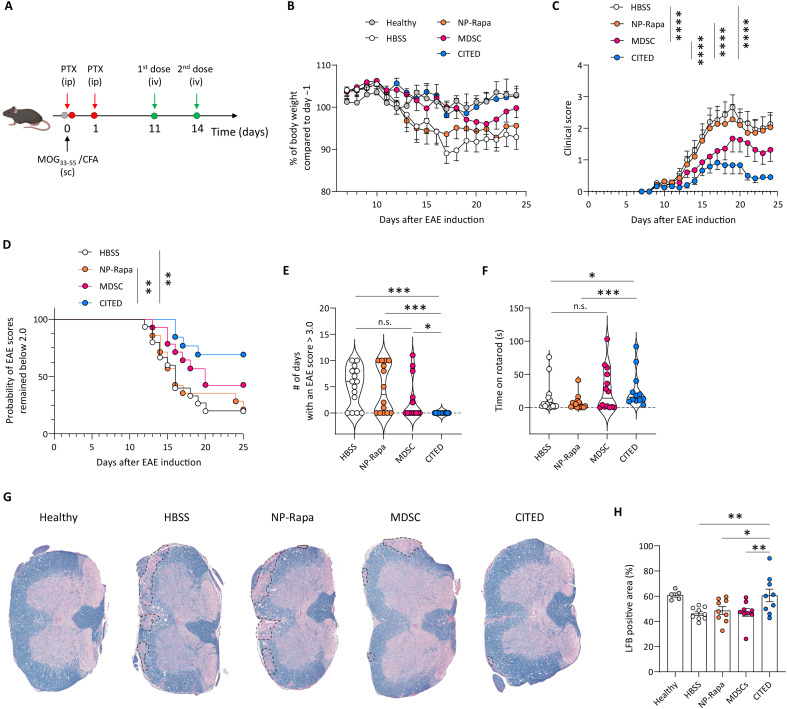
CITED inhibits disease progression in an EAE model. (**A**) Schematic showing the schedule for the therapeutic efficacy study. Created in BioRender: Z. Zhao (2025); https://BioRender.com/2tie67h. (**B**) Body weight change of EAE mice following different treatments. (**C**) Time-course changes of clinical scores for the EAE mice treated by different formulations. (**D**) Probability of clinical scores remaining <2.0 in different treatment groups. (**E**) Number of days with an EAE score of >3.0 for mice treated by different formulations. (**F**) Time (seconds) of mice remaining on a rotarod following different treatments on day 20 after disease induction. (**G**) Representative images of mouse spinal cord stained with Luxol fast blue (LFB) and fast red. The dotted circles indicate damaged area in the white matter. (**H**) Percentage of LFB-positive areas in the white matter of spinal cord. For (B) to (F), *n* = 5 biologically independent animals for the healthy group; *n* = 12 to 14 biologically independent animals for the other groups. For (H), *n* = 5 biologically independent animals for the healthy group; *n* = 9 biologically independent animals for the other groups. Data in (B), (C), (E), (F), and (H) are presented as means ± SEM. For (C), statistical analysis was conducted by two-way ANOVA with Dunnett’s test: ****P < 0.0001. For (D), statistical analysis was conducted by Mantel-Cox test: **P < 0.01. For (E) and (F), statistical analysis was conducted by Mann-Whitney test: *P < 0.05; ***P < 0.001. For (H), statistical analysis was conducted by one-way ANOVA with Dunnett’s test: *P < 0.05; **P < 0.01.

Building on findings that CITED efficiently migrates to and accumulates in damaged CNS tissue, we next sought to evaluate its therapeutic efficacy in an established myelin oligodendrocyte glycoprotein (MOG)_35–55_ vaccination–induced EAE model. In this model, mice were treated after disease onset to assess the therapeutic efficacy of CITED (Fig. 4A). We used various parameters to assess treatment efficacy, including body weight loss, clinical scores, motor coordination performance, and myelin damage. Notably, Hank’s balanced salt solution (HBSS) was used to resuspend the cells prior to in vivo injection, as it better preserves cell viability compared to similar buffers such as saline and PBS. Accordingly, HBSS was used as the vesicle-negative control in the in vivo studies. HBSS-treated and NP-Rapa–treated mice showed similar patterns of body weight loss, with a sharp decline starting on day 13 (Fig. 4B). MDSC-treated mice showed a delayed onset of weight loss but also experienced a marked decline beginning on day 19. In contrast, mice in the CITED group showed no significant weight loss throughout the entire study period.

We also monitored clinical scores to assess the severity of disease progression (Fig. 4C). Compared to the HBSS-treated mice, MDSC-treated mice had significantly lower clinical scores, while the NP-Rapa treatment did not yield a notable benefit. Throughout the study, CITED-treated mice maintained an average clinical score of less than 1, which indicates only a limp tail with no abnormalities in leg movement. On day 24, 84.6% of the CITED-treated mice had an EAE score below 0.5, showing far greater therapeutic efficacy than in the HBSS, NP-Rapa, and MDSC groups (26.7, 28.6, and 50.0% of mice), respectively. In addition, throughout the entire study period, 69.2% of mice in the CITED group never reached a score of 2.0 or above (Fig. 4D). Although MDSC treatment also led to a higher percentage of mice maintaining a clinical score below 2.0 compared to the HBSS treatment, its effect was less potent than that of CITED. Furthermore, we recorded the total number of days each mouse exhibited a clinical score of 3.0 or above, which indicates a limp tail and paralysis of at least two legs. While the MDSC treatment did not show significant benefits, none of the CITED-treated mice reached a score of 3.0 (Fig. 4E).

We conducted a rotarod test (*46*) on day 20 to assess whether CITED could improve motor coordination of EAE mice (Fig. 4F). Compared to the HBSS treatment, NP-Rapa did not increase the time mice remained on the rotarod. MDSC treatment alone marginally improved motor coordination in a subset of treated mice. In contrast, CITED markedly increased rotarod performance compared to the HBSS treatment, indicating its robust efficacy in inhibiting disease progression. In addition, we conducted a histological analysis where mouse spinal cord samples were stained with Luxol fast blue (LFB) to detect myelin damage, a key pathological feature of MS (*1*, *47*). CITED showed significantly better efficacy in reducing myelin damage in the white matter compared to HBSS, NP-Rapa, and MDSC alone (Fig. 4, G and H). Furthermore, immunohistology staining revealed that CITED led to a significantly higher myelin basic protein (MBP) expression in the spinal cord than the HBSS treatment (fig. S14, A and B), further demonstrating CITED’s robust efficacy in preventing demyelination. Overall, our results demonstrate that CITED effectively inhibits EAE progression and promotes recovery.

Next, we evaluated the safety of CITED through blood chemistry and histological analyses. Blood chemistry results showed that CITED did not induce abnormal changes in liver function– or kidney function–related markers (fig. S15A). Consistently, histological analysis revealed no detectable damage to major organs caused by CITED (fig. S15B). These data collectively support the overall safety of CITED.

### CITED modulates the adaptive and innate immune environment in the CNS to promote immune homeostasis restoration

**Fig. 5.**
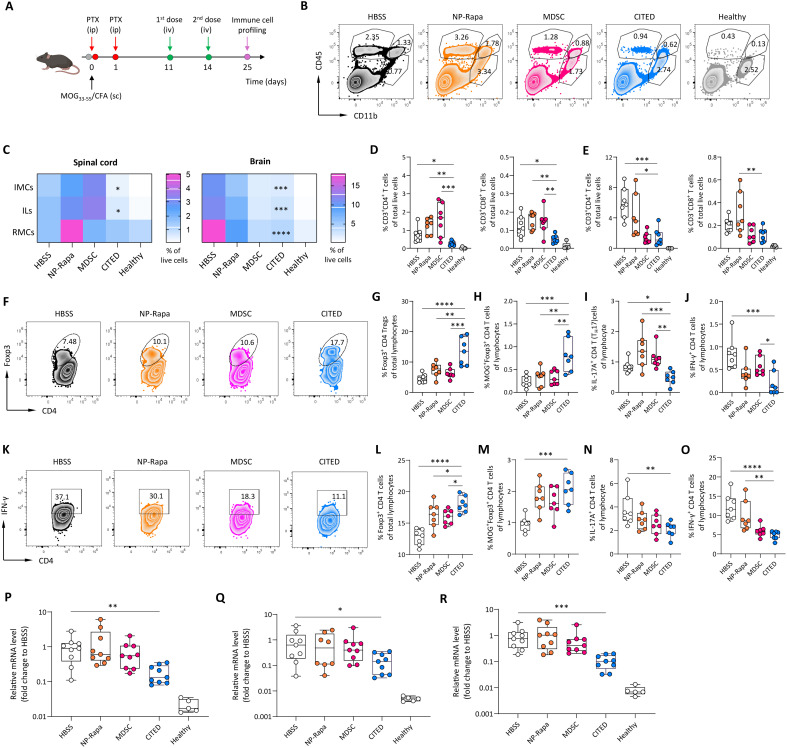
CITED modulates the adaptive immune environment of the spinal cord and brain to favor EAE resolution. (**A**) Schematic showing the study design. Created in BioRender: Z. Zhao (2025); https://BioRender.com/2tie67h. (**B** and **C**) Infiltrating and resident immune cells in the spinal cord and brain. (B) Representative flow cytometry plot showing different immune cell populations in the spinal cord. (C) Heatmap showing the relative number of infiltrating myeloid cells (IMCs), infiltrating lymphocytes (ILs), and resident myeloid cells (RMCs) in the spinal cord and brain. (**D** and **E**) Relative number of CD4 and CD8 T cells in the spinal cord (D) and brain (E). (**F** to **J**) CD4 T cell phenotypes in the spinal cord. [(F) and (G)] Representative flow cytometry plot (F) and relative number (G) of T_reg_ cells in the spinal cord. [(H) to (J)] Relative number of MOG_38–49_–specific T_reg_ cells (H), T_H_17 cells (I), and IFN-γ–expressing T_H_1 cells (J) in the spinal cord. (**K** to **O**) CD4 T cell phenotypes in the brain. (K) Representative flow plot of IFN-γ–expressing T_H_1 cells in the brain (pregated on CD4 T cells). [(L) to (O)] Relative number of T_reg_ cells (L), MOG_38–49_–specific T_reg_ cells (M), T_H_17 cells (N), and IFN-γ–expressing T_H_1 cells (O) in the brain. (**P** to **R**) qPCR quantification of the mRNA levels of IFN-γ (P), IL-1β (Q), and tumor necrosis factor–α (TNF-α) (R) in the spinal cord. For (C) to (O), *n* = 5 to 7 biologically independent animals. For (P) to (R), *n* = 5 to 9 biologically independent animals for the other groups. Data in (D), (E), (G) to (J), and (L) to (R) are presented as means ± SEM. For (C) to (E), (G) to (J), and (L) to (O), one-way ANOVA with Dunnett’s test: *P < 0.05; **P < 0.01; ***P < 0.001; ****P < 0.0001; for (C), statistical comparison was for CITED versus HBSS. For (P) to (R), one-way ANOVA with Kruskal-Wallis test: *P < 0.05; **P < 0.01; ***P < 0.001.

We conducted an immune cell profiling study to measure CITED’s ability to reprogram the immune microenvironment in the spinal cord and brain (Fig. 5A). Healthy CNS is an immune-privileged environment, and lymphocyte and myeloid cell infiltration is typically low. Massive infiltration of these cells into the spinal cord and brain is a hallmark of autoimmune activation in MS (*1*, *2*, *48*). Overall, CITED treatment significantly reduced the number of infiltrating myeloid cells and lymphocytes in the spinal cord and brain (Fig. 5, B and C, and figs. S16, A and B, and S18A), whereas MDSC or NP-Rapa treatments showed less benefit. Specifically, CITED demonstrated a 3.7- to 8.8-fold and a 2.7- to 5.6-fold greater efficacy in reducing myeloid cell and lymphocyte infiltration into the spinal cord, respectively, compared to HBSS, NP-Rapa, and MDSC treatments. Further analysis revealed that CITED markedly decreased spinal cord infiltration of various immune cells to levels closer to those seen in healthy mice, such as CD4 T cells (Fig. 5D), CD8 T cells (Fig. 5D), and B cells (fig. S17). Similar results were observed in the brain where CITED treatment reduced infiltration of various immune cells (Fig. 5E and fig. S18B). Notably, MDSC treatment alone significantly reduced immune cell infiltration in the brain but showed no significant effect in the spinal cord.

Beyond reducing immune cell infiltration into the CNS at the global level, CITED also effectively modulated both adaptive and innate immune cell phenotypes. Due to the low number of infiltrating cells into the spinal cord and brain of healthy mice, which was not sufficient for further phenotype analysis, we did not include the healthy group in this analysis. Specifically, for CD4 T cell phenotypes, CITED treatment significantly increased the relative frequency of T_reg_ cells and antigen-specific T_reg_ cells in the spinal cord, resulting in a 1.8- to 2.9-fold and 2.4- to 3.5-fold increase, respectively, compared to the other treatments (Fig. 5, F to H). In addition, compared to the HBSS treatment, CITED significantly reduced the frequency of T_H_1 and T_H_17 cells in the spinal cord (Fig. 5, I and J), two T cell phenotypes known to be up-regulated in MS and contribute to disease progression (*49*, *50*). Notably, the shifting of CD4 T cells toward T_reg_ cells promoted by CITED may benefit long-term disease control, as T_reg_ cells, particularly antigen-specific T_reg_ cells, contribute to sustained immunotolerance in autoimmune diseases (*51*, *52*). Similarly, in the brain of EAE mice, CITED treatment also significantly increased T_reg_ cells and antigen-specific T_reg_ cells while simultaneously reducing T_H_1 and T_H_17 cells (Fig. 5, K to O). Furthermore, we found that CITED significantly down-regulated the expression of proinflammatory cytokines in the spinal cord of EAE mice, such as tumor necrosis factor–α (TNF-α), IL-1β, and IFN-γ (Fig. 5, P to R), supporting the finding that CITED significantly alleviated the CNS inflammation.

**Fig. 6.**
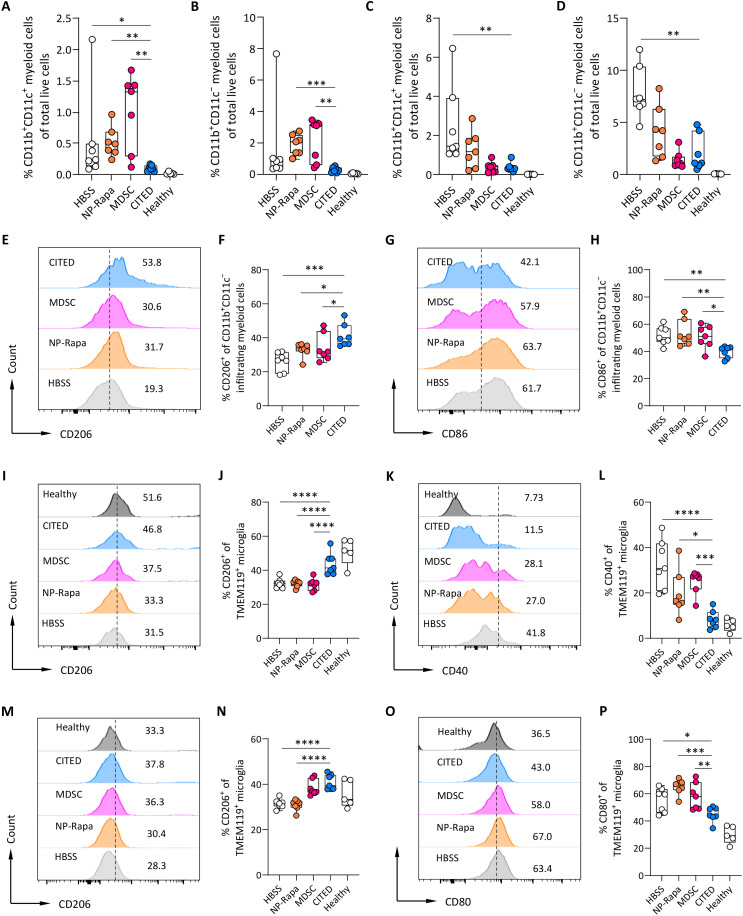
CITED modulates the innate immune environment of the CNS to favor EAE resolution. (**A** to **D**) Number of different infiltrating myeloid cells in the CNS following different treatments. [(A) and (B)] Percentage of infiltrating CD11b^+^CD11c^+^ (A) and CD11b^+^CD11c^−^ (B) myeloid cells in the spinal cord. (C and D) Percentage of infiltrating CD11b^+^CD11c^+^ (C) and CD11b^+^CD11c^−^ (D) myeloid cells in the brain. (**E** to **L**) Phenotype of myeloid cells in the CNS. (E to H) Representative flow plots (E and G) and quantitative analysis [(F) and (H)] of CD206^+^ (M2-like) and CD86^+^ (M1-like) infiltrating myeloid cells in the spinal cord. (I to L) Representative flow plots [(I) and (K)] and quantitative analysis [(J) and (L)] of CD206^+^ (M2-like) and CD40^+^ (M1-like) resident microglia in the spinal cord. (**M** to **P**) Phenotype of resident microglia in the brain. Representative flow plots [(M) and (O)] and quantitative analysis of CD206^+^ (N) and CD80^+^ (P) resident microglia in the brain. For (A) to (P), *n* = 5 biologically independent animals for the healthy group; *n* = 7 biologically independent animals for the other groups. Data in (A) to (D), (F), (H), (J), (L), (N), and (P) are presented as means ± SEM. For (A) to (D), statistical analysis was conducted by one-way ANOVA with Kruskal-Wallis test: *P < 0.05; **P < 0.01; ***P < 0.001. For (F), (H), (J), (L), (N), and (P), statistical analysis was conducted by one-way ANOVA with Dunnett’s test: *P < 0.05; **P < 0.01; ***P < 0.001; ****P < 0.0001.

Next, we evaluated the infiltration of myeloid cell subtypes in the spinal cord and brain following different treatments. CITED significantly reduced the infiltration of CD11b^+^CD11c^−^ and CD11b^+^CD11c^+^ myeloid cells in both the spinal cord and brain, while MDSC or Rapa NP alone showed only modest effect in the brain (Fig. 6, A to D). Subsequently, we assessed the phenotype of infiltrating myeloid cells and resident microglia in the CNS. In the spinal cord, while the MDSC treatment alone did not show significant effect, CITED treatment significantly promoted the anti-inflammatory (M2-like) phenotype of infiltrating CD11b^+^CD11c^−^ myeloid cells, resulting in a 1.6-fold increase in CD206^+^ anti-inflammatory M2-like cells and a 24% reduction in CD86^+^ proinflammatory M1-like cells compared to HBSS treatment (Fig. 6, E to H). Similarly, CITED significantly modulated the phenotype of resident microglia, with a 1.3- to 1.4-fold increase in CD206^+^ microglia and a 57.9 to 74.6% reduction in CD40^+^ microglia compared to other treatment groups (Fig. 6, I to L). In the brain, CITED did not significantly alter the phenotype of infiltrating CD11b^+^CD11c^−^ myeloid cells (fig. S18, C and D) but showed a robust effect in promoting the anti-inflammatory phenotype of resident microglia (Fig. 6, M to P). Notably, MDSC treatment alone significantly up-regulated CD206^+^ microglia but had no significant effect on CD80^+^ microglia in the brain. Collectively, our findings demonstrate that CITED show a robust capability to modulate both the innate and adaptive immune environment in the CNS during EAE through multifaceted mechanisms, including reducing overall immune cell infiltration, promoting T_reg_ cell accumulation, and modulating the phenotype of myeloid cells.

We also performed immune cell profiling on the spleen and spinal cord–draining lymph nodes (SCDLNs) to assess CITED’s impact on the systemic immune response. Compared to the HBSS treatment, CITED did not significantly alter the relative frequency of general T cell and myeloid cell populations in either the spleen or SCDLNs, except for a marked increase in Gr1^+^CD11b^+^ MDSCs (figs. S19, A to F, and S20, A to F). While the overall frequencies of T_H_1 cells, T_H_17 cells, and T_reg_ cells were similar between the CITED and HBSS groups (figs. S19, G to I, and S20, G to I), CITED treatment resulted in a higher percentage of antigen-specific T_reg_ cells in both the spleen and SCDLNs of EAE mice (figs. S19, J and K, and S20J). Moreover, further analysis revealed that following the CITED treatment, T_reg_ cells in the spleen and SCDLNs expressed elevated levels of immune checkpoint markers, such as PD-1 and CTLA-4 (figs. S19, L to O, and S20, K and L). Antigen-specific T_reg_ cells in the spleen and SCDLNs can serve as a reservoir and sustain to modulate the disrupted immune tolerance in the CNS.

In addition, we assessed the immune cell profiles in the lungs. Although CITED accumulated substantially in the lungs, it did not significantly alter the numbers of major lung immune cell populations including CD4 and CD8 T cells, CD4 T cell subsets, B cells, alveolar macrophages, and various myeloid cell subtypes, compared with the control HBSS treatment (fig. S21). This is likely because MDSC-mediated immunosuppression depends on both the activation status of target cells (e.g., T cells) and the inflammatory state of the surrounding tissue environment. MDSCs primarily suppress activated T cells in inflammatory environments, while nonactivated T cells in noninflammatory settings are relatively spared (*24*–*26*). Because the lungs are minimally inflamed in EAE, the suppressive effect of MDSCs/CITED in the lungs is expected to be limited.

## DISCUSSION

In this work, we report an engineered MDSC-based approach, CITED, for targeted immunotherapy of MS. CITED hinges on two key mechanisms of action to achieve precise and multimodal immune reprogramming in CNS. First, CITED harnesses the unique immunosuppressive properties of MDSCs, enhanced by the surface conjugation of rapamycin NPs, to function as an engineered cell therapy for immunomodulation. Second, CITED takes advantage of the intrinsic inflammation-homing ability of MDSCs to improve CNS-targeted accumulation, enhances rapamycin NP delivery to the CNS, and facilitates localized CNS-specific immunomodulation. In addition, the decoration of MDSC surface with rapamycin NPs enhances both mechanisms by amplifying the immunomodulatory functions of MDSCs and improving their migration capabilities. Our results demonstrate that by integrating these two mechanisms, CITED enables CNS-targeted, multimodal reprogramming of both innate and adaptive immune responses, restoring immune tolerance and effectively inhibiting MS disease progression. Notably, the dose of rapamycin used in our animal studies (8 μg/kg) was lower than that typically used in conventional rapamycin therapies, which are administered in the milligram per kilogram range. In CITED, rapamycin primarily enhances the immunomodulatory functions of MDSCs rather than mainly resolving inflammation through its direct anti-inflammatory effects.

The backbone of the CITED approach is the MDSC, a unique type of myeloid cells with intrinsic immunosuppressive activity. While the immunosuppressive functions of MDSCs in cancer have been relatively well elucidated (*53*), their role as a therapy in autoimmune diseases remains understudied. Insights from cancer-related studies, along with emerging evidence from autoimmune studies, suggest that MDSCs can modulate both innate and adaptive immunity through multifaceted mechanisms involving both cell contact–dependent and cell contact–independent pathways (*27*). However, a key challenge for their therapeutic use lies in their transient and suboptimal immunosuppressive activity, which is highly sensitive to changes of the surrounding disease immune environment. By leveraging surface-conjugated rapamycin NPs to enhance MDSC’s immunosuppressive function, CITED provides an effective approach for engineering MDSCs as potent cell therapies for autoimmune diseases such as MS. Our data demonstrate that while MDSCs or rapamycin NPs alone show limited efficacy in immune reprogramming or disease progression inhibition, the integration of both components via click chemistry–mediated linking enhances the immunomodulatory effects and therapeutic efficacy.

The effect of rapamycin NPs on MDSCs is most likely derived from both the rapamycin and the degradation product of PLGA. Notably, our data indicate that conjugating blank PLGA NPs to MDSCs also improves MDSC immunosuppressive function, although less effectively than rapamycin NPs. Prior studies have shown that lactic acid, a PLGA degradation product, can affect intracellular metabolic pathways, up-regulate immune checkpoint expression on myeloid cells such as PD-L1, and enhance MDSC’s immunosuppressive function (*39*, *40*). Our data are in line with these reports. In addition, rapamycin, an inhibitor for the mammalian target of rapamycin, has also been reported to enhance the immunosuppression function of MDSCs via modulating the NF-κB signaling pathway (*32*). By conjugating rapamycin-PLGA NPs to MDSCs as “backpacks,” CITED enables sustained release of rapamycin and lactic acid, resulting in prolonged enhancement of MDSC’s immunosuppressive activity. Our unbiased transcriptomic analysis demonstrated that the decoration with blank PLGA NP itself can augment the chemotactic gene expression program and that the addition of rapamycin further reprograms the expression of genes related to the regulation of immune responses and chemotaxis. CITED, therefore, generates cells that can rapidly migrate to inflammatory sites. We found that NP and rapamycin both down-regulate cell cycle progression, which could be indicative of reduced differentiation of the cells and also underscores the safety of CITED because there is less risk of unfettered cell proliferation after cell delivery.

A key feature of CITED is its capability to efficiently migrate to the inflamed CNS through an active trafficking mechanism, which addresses a major challenge in MS therapy. This targeted trafficking is driven by the innate chemotactic properties of MDSCs and the elevated expression of chemokine receptors, such as CCR2 and CX3CR1, following rapamycin NP conjugation. CITED achieved significantly higher accumulation of NPs or injected cells in the CNS compared to free NPs or MDSCs alone, demonstrating its robust ability to cross the blood-brain barrier and enhance the delivery of therapeutic agents directly to the inflammation sites. The robust therapeutic efficacy of CITED in the EAE model is attributed to its multifaceted immunomodulatory mechanisms. CITED treatment significantly reduced infiltration of immune cells into the CNS, rebalanced the proinflammatory and regulatory CD4 T cell phenotypes, and polarized infiltrating myeloid cells and resident microglia to anti-inflammatory phenotypes. CITED’s ability to modulate both adaptive and innate immune responses underscores its potential for sustained disease inhibition. By increasing the number of T_reg_ cells, including antigen-specific T_reg_ cells, and reducing proinflammatory T cell subsets such as T_H_1 and T_H_17 cells, CITED promotes the restoration of immune tolerance in the CNS. The polarization of resident microglia and infiltrating myeloid cells toward an anti-inflammatory phenotype further supports the resolution of neuroinflammation. These immunomodulatory effects were localized to the CNS, with minimal impact on systemic immune cell populations, highlighting the specificity of CITED. Notably, our in vivo data showed that CITED increased the frequency of T_reg_ cells in the brain and spinal cord. This increase was most likely due to the local expansion of preexisting T_reg_ cells within the CNS rather than de novo generation, as de novo generation from naive CD4 T cells typically occurs in secondary lymphoid organs. Local expansion of T_reg_ cells in the CNS in EAE has been previously reported (*54*). However, our current data could not exclude the possibility that CITED promoted the de novo generation of T_reg_ cells, especially antigen-specific T_reg_ cells, in secondary lymphoid organs, which subsequently infiltrated the CNS. Future studies are needed to determine whether CITED primarily induces de novo T_reg_ cell generation or expands resident T_reg_ cell populations. Notably, compared to other promising investigational cell therapies for MS, such as chimeric antigen receptor T cells that primarily target a single cell population (in most cases, B cells) for depletion (*55*), CITED may offer a more comprehensive approach due to its multimodal immunomodulatory capability, which allows it to address multiple pathological pathways.

It is important to note that, while we only demonstrated CITED’s therapeutic potential in MS, its modular design offers the flexibility to incorporate other rationally selected payloads, allowing potential adaptation for other inflammatory diseases and autoimmune disorders where immune homeostasis is disturbed. However, the broader applicability of CITED needs to be further investigated in future studies. Notably, CITED does not require any ex vivo genetic engineering, which mitigates some of the manufacturing challenges associated with conventional cell therapies. Nonetheless, the complexity of the CITED approach (e.g., the source of MDSCs) needs to be further considered for clinical translation. Isolating an adequate number of native MDSCs from a patient’s blood may be challenging. A more feasible approach may involve producing MDSCs through differentiation from bone marrow cells or by leveraging induced pluripotent stem cell–based methods. In summary, CITED represents a comprehensive approach for multimodal targeted immunomodulation and provides a broadly effective platform for developing effective immunotherapies for MS and potentially other autoimmune disorders.

## MATERIALS AND METHODS

### Materials

Poly(lactide-*co*-glycolide) (PLGA, Resomer RG 653 H) was purchased from Sigma-Aldrich (St. Louis, MO). Poly(lactide-*co*-glycolide)-poly(ethylene glycol) with dibenzocyclooctyne [PLGA-PEG-DBCO; molecular weight (MW): 20 kDa/5 kDa] and poly(lactide-*co*-glycolide)-*b*-poly(ethylene glycol) methyl ether (mPEG-PLGA; MW: 2 kDa/20 kDa) were purchased from Nanosoft Polymers (Lewisville, NC). Rapamycin was purchased from MedChemExpress (Monmouth, NY). Penicillin/streptomycin was obtained from Cytiva (Marlborough, MA). RPMI-1640 and heat-inactivated FBS were purchased from Corning (Corning, NY). Recombinant murine IL-6 and recombinant murine GM-CSF were obtained from BioLegend (San Diego, CA). 2-Mercaptoethanol, Hepes buffer, ACK buffer, sodium pyruvate, 1,1′-dioctadecyl-3,3,3′,3′-tetramethylindodicarbocyanine (DiD), 3,3′-dioctadecyloxacarbocyanine perchlorate (DiO), 1,1′-dioctadecyl-3,3,3′,3′-tetramethylindocarbocyanine perchlorate (DiI), 1,1′-dioctadecyl-3,3,3′,3′-tetramethylindotricarbocyanine iodide (DiR), IVISense 680 fluorescent cell labeling dye, Hoechst 33342, and CellTrace Violet were purchased from Thermo Fisher Scientific (Waltham, MA). DBCO–PEG_3_–fluorescein isothiocyanate (FITC) was purchased from Conju-Probe (San Diego, CA). *N*-azidoacetylgalactosamine-tetraacylated (Ac4GalNAz) was purchased from Vector Labs (Newark, CA). Multi-tissue dissociation kits and debris removal solution were purchased from Miltenyi Biotec (Gaithersburg, MD). MBP (D8X4Q) XP Rabbit Monoclonal Antibody was purchased from Cell Signaling (Beverly, MA). EasySep Mouse MDSC (CD11b^+^Gr1^+^) Isolation Kit and EasySep Mouse CD4^+^ T Cell Isolation Kit were purchased from STEMCELL Technologies (Vancouver, BC, Canada). High-Capacity cDNA Reverse Transcription Kit was purchased from Applied Biosystems (Waltham, MA). AceQ Universal SYBR qPCR Master Mix was purchased from Vazyme (San Diego, CA).

### Preparation and characterization of rapamycin NPs

Rapamycin-loaded NPs were prepared using a nanoprecipitation method. Briefly, 3 mg of PLGA (Resomer RG 653 H), 2 mg of mPEG-PLGA (MW: 2 kDa/20 kDa), and 1 mg of PLGA-PEG-DBCO (MW: 20 kDa/5 kDa) were dissolved in 300 μl of acetone. For rapamycin-loaded NPs, rapamycin constituting 14.5% of the polymers (w/w) was dissolved in acetone along with the polymers. The acetone mixture was then added dropwise into 3 ml of deionized (DI) water under constant stirring. The mixture was left in a fume hood overnight to ensure the complete evaporation of the organic solvent. NPs were washed three times with ultrapure water before being characterized for physicochemical properties. The hydrodynamic diameter and zeta potential of NPs were determined using a dynamic light scattering method (Malvern Nano-ZS Zetasizer). The morphology of NPs was characterized by SEM (JEOL JSM-IT500HR). The release profile of rapamycin from NPs was measured using a dialysis method against PBS using a dialysis membrane with a molecular cutoff of 10 kDa, and rapamycin was quantified using a Shimadzu ultrahigh-performance liquid chromatography (UHPLC) equipped with an ACQUITY UPLC BEH C18 Column (130 Å, 1.7 μm, 2.1 mm by 50 mm, Waters), using a 5 to 95% gradient mobile phase consisting of acetonitrile (0.1% formic acid) in water (0.1% formic acid). Rapamycin release from CITED in PBS containing 10% FBS was measured using a similar method. Rapamycin was quantified using a Shimadzu 8060 triple quadrupole mass spectrometer interfaced to a Nexera X2 LC-30AD solvent delivery system, equipped with an Agilent SB-CN cyano column (2.1 mm by 50 mm, 1.8 μm), using a mobile phase consisting of 5 mM ammonium acetate/0.1% formic acid (solvent A) and acetonitrile/0.1% formic acid (solvent B). The separation gradient was 43 to 98% solvent B over 3 min at a flow rate of 0.3 ml/min.

### MDSC culture

MDSCs were cultured from mouse bone marrow using IL-6 and GM-CSF as growth factors (*33*). Briefly, bone marrow was obtained from the tibias, femurs, and humeri of freshly euthanized female C57BL/6 mice (6 to 10 weeks of age). The harvested bone marrow cells were seeded into a 250 mm–by–20 mm petri dish at a concentration of 2.5 × 10^5^ cells/ml and cultured in RPMI-1640 medium supplemented with 10% FBS, 1% penicillin/streptomycin, 50 μM 2-mercaptoethanol, IL-6 (40 ng/ml), and GM-CSF (40 ng/ml). Nonadherent cells were harvested on day 3. To induce azido groups on the surface of MDSCs, Ac4GalNAz was added to the cell culture at a concentration of 50 μM on day 1 to obtain MDSC-azido. Harvested MDSCs were purified using an EasySep Mouse MDSC (CD11b^+^Gr1^+^) Isolation Kit (STEMCELL Technologies). To verify the presence of azido functional groups on MDSCs, MDSC-azido was resuspended in 3 ml of PBS at a concentration of 1 × 10^6^ cells/ml. The cells were then incubated with 2 μl of DBCO-PEG_3_-FITC (4 mg/ml in dimethyl sulfoxide) in the dark at room temperature for 30 min. The cells were washed with PBS three times and imaged using a fluorescence microscope (EVOS M5000 Imaging System, Thermo Fisher Scientific) or analyzed by flow cytometry (CytoFLEX, Beckman Counter).

### Conjugation of NPs to MDSCs for CITED preparation

NPs were conjugated to MDSCs using an azido-DBCO click chemistry–based method. In brief, MDSC-azido was resuspended in PBS containing 0.2% bovine serum albumin (BSA) at a concentration of 2 × 10^7^ cells/ml. Separately, 1 mg of NPs was resuspended in 75 μl of PBS containing 0.2% BSA. MDSCs and DBCO/PEG/PLGA NPs were mixed and incubated under continuous rotation on a revolver at 37°C for 1 hour. Conjugated MDSCs were subsequently washed three times with PBS containing 0.2% BSA to remove unbound NPs. To quantify the number of NPs on MDSCs, NPs were labeled with DiD and the number of NPs on cells were quantified using flow cytometry. In addition, the presence of NPs on MDSCs was characterized by CLSM and SEM. The amount of rapamycin carried by MDSCs was quantified using UHPLC according to the method described above.

### T cell proliferation inhibition assay

Mouse spleen was isolated from freshly euthanized female C57BL/6 mice (6 to 10 weeks of age) and mechanically disrupted, followed by passing the tissue through a 70-μm cell strainer to obtain a single-cell suspension. Red blood cells (RBCs) in the cell suspension were removed using ACK lysis buffer for 3 min on ice. After RBC lysis, splenocytes were washed with sterile PBS, labeled with CellTrace Violet, and prepared for the T cell inhibition study. In brief, MDSCs were cultured and prepared for coculture with CellTrace Violet–labeled splenocytes at varying ratios in RPMI-1640 medium supplemented with 10% FBS, 50 μM 2-mercaptoethanol, anti-mouse CD3 antibody (2.5 μg/ml; clone #145-2C11, BioLegend), and anti-mouse CD28 antibody (2.5 μg/ml; clone #E18, BioLegend). On day 3, fresh medium supplemented with 2-mercaptoethanol and antibodies was added to the culture. On day 5, the cell mixture was collected, stained with antibodies against CD4 (FITC, catalog #100510, clone #RM4-5, BioLegend) and CD8 (APC, catalog #162306, clone #S18018E, BioLegend), and analyzed for T cell proliferation by flow cytometry (CytoFLEX, Beckman Counter).

### CD4 T cell phenotype assay

Mouse CD4 T cells were collected from mouse spleens from freshly euthanized female C57BL/6 mice (6 to 10 weeks of age) using a murine CD4 T cell isolation kit (STEMCELL Technologies). MDSCs were cultured according to the method described before and prepared for coculture with CD4 T cells at varying ratios in RPMI-1640 medium supplemented with 10% FBS, 50 μM 2-mercaptoethanol, anti-mouse CD3 antibody (2.5 μg/ml; clone #145-2C11, BioLegend), and anti-mouse CD28 antibody (2.5 μg/ml; clone #E18, BioLegend). Fresh medium containing 2-mercaptoethanol and antibodies was added on day 3. On day 5, the cell mixture was collected, stained with antibodies against CD3 (Spark Blue 550, catalog #100260, clone #17A2, BioLegend), CD4 (Spark UV387, catalog #100492, clone #GK1.5, BioLegend), CD25 (APC/Fire810, catalog #102076, clone #PC61, BioLegend), Foxp3 (Alexa Fluor 647, catalog #126408, clone #FM-14, BioLegend), IFN-γ (APC/Fire750, catalog #505860, clone #XMG1.2, BioLegend), and IL-17A (BV785, catalog #506928, clone #TC11-18H10.1, BioLegend), and analyzed using flow cytometry (Aurora, Cytek).

### RNA-seq studies

The transcriptomes of MDSCs and MDSCs conjugated with NPs were assessed using bulk RNA-seq. Fresh MDSC, MDSC conjugated with blank NP, and MDSC conjugated with NP-Rapa NPs (CITED) were cultured in complete RPMI-1640 medium supplemented with IL-6 (40 ng/ml) for 24 hours. Total RNA was extracted from cells using the RNeasy Mini Kit (QIAGEN) in accordance with the manufacturer’s protocol. Briefly, 5 million cells were homogenized in cell lysis buffer, and the lysate was passed through a spin column to capture RNA. RNA was eluted in ribonuclease (RNase)–free water after washing. All the procedures were performed at 4°C to ensure RNA integrity. The quantity and purity of RNA were evaluated using a NanoDrop 2000 (Thermo Fisher Scientific). Extracted RNAs were stored at −80°C for subsequent RNA-seq analysis. Quantification analysis, RNA sample quality control, mRNA library preparation (poly A enrichment), and RNA-seq using the NovaSeq X Plus Series (PE150) were conducted at Novogen using standard protocols. RNA-seq data analysis was performed according to the method detailed in the Supplementary Materials.

### Biodistribution studies

Biodistribution of CITED and control formulations was tested in a MOG_35–55_–induced EAE model. All the animal experiments were performed in compliance with National Institutes of Health and institutional guidelines. All animal procedures were conducted according to approved protocols by the Institutional Animal Care and Use Committee at the University of Illinois, Chicago (21-098, 24-085). The EAE model was induced according to a protocol provided by the manufacturer (Hooke Laboratories). In brief, female C57BL/6 mice (10 weeks of age, the Jackson Laboratory) were housed in the animal facility for at least 1 week before disease induction. To induce EAE, on day 0, MOG_35–55_/complete Freund’s adjuvant (CFA) emulsion (~1 mg of MOG_35–55_ per milliliter of emulsion) was subcutaneously administered to two sites (0.1 ml per site). Pertussis toxin (PTX) was then intraperitoneally injected 2 and 24 hours later (120 ng per dose per mouse). Mice were kept minimally disturbed during the EAE studies. The development of EAE was monitored, and clinical scores and body weight of the mice were recorded daily. For the biodistribution study, on day 14 after MOG_35–55_ injection, labeled CITED or control formulations in which MDSCs and NPs were labeled with IVISense 680 and DiI or DiR, respectively, were resuspended in 150 μl of HBSS and injected into EAE mice via intravenous injection. Mouse organs were collected 20 and 72 hours after formulation injection and imaged using Lago X (Spectral Instruments Imaging). In a separate study for imaging biodistribution using confocal fluorescence microscopy, the brain and lumbar spinal cord were collected 20 hours after intravenous injection of CITED and fixed in 4% paraformaldehyde solution for 15 min at room temperature, followed by PBS washing. Fixed tissues were embedded into a 2% agarose gel (dissolved in DI water, LE Quick Dissolve Agarose, GeneMate). Embedded tissues were sectioned to 400-μm thickness using a vibratome (VT1200 S, Leica). The sectioned tissues were stained with 4,6-diamidino-2-phenylindole (DAPI) for nucleus visualization. An optical clearing method was used to enhance the imaging depth of the tissue samples. To achieve this, tissues were incubated in ascending concentrations of d-fructose solutions (20, 50, 80, and 100%, w/v, in 10 mM PBS) at room temperature. Fluorescence images were acquired using an upright confocal microscope (Caliber I.D., RS-G4).

To evaluate the capability of CITED to enhance NP and rapamycin delivery to the brain and spinal cord, DiR was coloaded into NP-Rapa to prepare either free NP-Rapa or CITED formulations. A single dose of CITED (5 million cells) or NP-Rapa (equivalent NP dose to that in CITED) was intravenously injected into EAE mice on day 14. Brain and spinal cord were collected at 24, 48, or 96 hours and imaged using the Lago X system (Spectral Instruments Imaging). Tissues were then homogenized in 50% acetonitrile to extract rapamycin. Ecumicin was used as an internal standard and added to the tissue homogenate at a 1:2 (v/v) ratio. The homogenates were then centrifuged at 20,000*g* for 15 min, and the supernatants were collected for LC/mass spectrometry analysis. Quantitative measurement of rapamycin concentration was carried out using positive ion electrospray ionization on a Shimadzu 8060 triple quadrupole mass spectrometer interfaced to a Nexera X2 LC-30AD solvent delivery system. Separations were carried out on an Agilent SB-CN cyano column (2.1 mm by 50 mm, 1.8 μm) with a mobile phase consisting of 5 mM ammonium acetate/0.1% formic acid (solvent A) and acetonitrile/0.1% formic acid (solvent B). The gradient was set from 43 to 98% solvent B over 3 min at a flow rate of 0.3 ml/min with the column thermostated at 40°C. Mass spectrometry data were acquired in the SRM mode using the transition 931.4 > 864.4 at a collision energy of 22 eV.

### Therapeutic efficacy studies

The therapeutic efficacy studies were conducted in the MOG_35–55_–induced EAE model, which was induced according to the same protocol described before. In this model, mice start to develop symptoms on day 9. On day 9, mice were randomized into different treatment groups. On days 11 and 14, mice were intravenously injected with CITED or control formulations including HBSS, free rapamycin NPs (8 μg/kg), free MDSCs (5 million), and CITED [5 million MDSCs, rapamycin (8 μg/kg)]. Clinical scores and body weight of mice were monitored daily. The clinical scores were determined using an established disease score rubric from Hooke Laboratories. On day 20, the motor coordination ability of EAE mice after different treatments was assessed via a rotarod assay (*46*). On day 25, the spinal cord of randomly selected mice in each treatment group was collected for histological staining and analysis according to methods detailed in the Supplementary Materials.

### Immune cell profiling studies

To evaluate CITED’s ability to modulate the immune responses in EAE mice, mice were treated according to the same schedule and dosing regimen as in the efficacy studies. On day 25, the brain, spinal cord, spleen, and SCDLNs (iliac and cervical lymph nodes) of mice were collected and processed into single-cell suspensions. Spleen and lymph nodes were processed by mechanically pushing the tissues through a 70-μm cell strainer, while the brain and spinal cord were processed using a Multi Tissue Dissociation Kit 1 (Miltenyi Biotech). The cells were washed with PBS and stained with antibodies at titrated concentrations. Antibodies were obtained from BioLegend, National Institutes of Health Tetramer Core, or Invitrogen, including the following (i) from BioLegend: anti-CD45 (Spark Blue 574, catalog #103184, clone #30-F11), anti-CD11b (PerCP/Fire806, catalog #101294, clone #M1/70), anti-CD11c (PerCP, catalog #117326, clone #N418), anti-F4/80 (BV510, catalog #123135, clone #BM8), anti-CD80 (BV650, catalog #104732, clone #16-10A1), anti-CD86 (AF700, catalog #105024, clone #GL-1), anti-MHCII (BV605, catalog #107639, clone #M5/114.15.2), anti-CD206 [(phycoerythrin) PE/Fire700], catalog #141741, clone #C068C2), anti-Gr1 (BV570, catalog #108431, clone #RB6-8C5), anti-CD40 (BV421, catalog #124641, clone #3/23), anti-CD3 (Spark Blue 550, catalog #100260, clone #17A2), anti-CD4 (Spark UV387, catalog #100492, clone #GK1.5), anti-CD8 (Pacific Blue, catalog #100725, clone #53-6.7), anti-Foxp3 (AF647, catalog #126408, clone #MF-14), anti-CD25 (APC/Fire810, catalog #102076, clone #PC61), anti-IFN-γ (APC/Fire750, catalog #505860, clone #XMG1.2), anti-IL17A (BV785, catalog #506928, clone #TC11-18H10.1), anti-CD19 (FITC, catalog #152403, clone #1D3/CD19), anti-PD-1 (PE/Cy5, catalog #135256, clone #29F.1A12), anti-PD-L1 (BV711, catalog #124319, clone #10F.9G2), anti-CTLA-4 (PE/Dazzle594, catalog #106318, clone #UC10-4B9), and Zombie UV (catalog #423108); (ii) from National Institutes of Health Tetramer Core: PE-conjugated MOG_38–49_ tetramer; and (iii) from Invitrogen: anti-TMEM119 (PE/Cy7, catalog #103184, clone #V3RT1GOsz). Stained cell samples were subsequently measured by flow cytometry (Aurora, Cytek). Details of the antibodies and their concentrations are further shown in tables S1 and S2.

In addition, the total RNA was extracted from spinal cord using the TRIzol Reagent (Thermo Fisher Scientific) following the manufacturer’s protocol. Briefly, samples were homogenized in cell lysis buffer and mixed with chloroform. After centrifugation, the RNA-containing phase was collected followed by precipitation using isopropanol and 70% ethanol. RNA pellets were eluted in RNase-free water after washing. The quantity and purity of RNA were assessed using NanoDrop 2000 (Thermo Fisher Scientific). For RT-qPCR, cDNA was synthesized from 2 μg of total RNA using the high-capacity cDNA reverse transcription kit. RT-qPCR was performed using the ViiA 7 Real-Time PCR System (Life Technologies) with an AceQ Universal SYBR qPCR Master Mix Kit. Primers were designed as shown in table S3, and the relative mRNA levels were normalized to the housekeeping gene *GAPDH*.

### Statistical analysis

All experiments were repeated at least two times. No samples or data were excluded from analysis. All data points described are biological replicates. For animal studies, sample size was determined based on power analysis. Statistical analyses were performed using GraphPad Prism 10. All data are presented as means ± standard error of the mean (SEM). Student’s *t* test, one-way analysis of variance (ANOVA), two-way ANOVA, or Mann-Whitney test were used to determine significance. *P* values represent different levels of significance: *P < 0.05; **P < 0.01; ***P < 0.001; ****P < 0.0001. All flow cytometry analyses were carried out using FlowJo 10 software.
