# Supplementary material for: Nanoparticle-boosted myeloid-derived suppressor cell therapy for immune reprogramming in multiple sclerosis
Source: Sci Adv. 2025 Oct 15;11(42):eady4135. doi: 10.1126/sciadv.ady4135 (PMC12526245; doi:10.1126/sciadv.ady4135)
Supplement: Supplementary file 1 — Supplementary Methods Figs. S1 to S27 Tables S1 to S3 [file sciadv.ady4135_sm.pdf]

Science Advances
AAAS

Supplementary Materials for

Nanoparticle-boosted myeloid-derived suppressor cell therapy for immune reprogramming in multiple sclerosis

Endong Zhang *et al.*

Corresponding author: Zongmin Zhao, zhaozm@uic.edu

Sci. Adv. **11**, eady4135 (2025)
DOI: 10.1126/sciadv.ady4135

This PDF file includes:

Supplementary Methods
Figs. S1 to S27
Tables S1 to S3

Supplementary Methods

MDSC co-culture with DCs. For bone marrow-derived dendritic cells (BMDCs) culturing, bone marrow was obtained from the tibias, femurs and humeri of freshly euthanized C57BL/6 mice. Cells were cultured in RPMI-1640 medium supplemented with 10% FBS, 20 ng/mL GM-CSF, and 50 μM 2-mercaptoethanol for 7 days to generate immature BMDCs. Fresh medium containing GM-CSF and 2-mercaptoethanol were added on days 3 and 6. On day 7, non-adherent cells (DCs) were then collected and stained with CellTrace™ CSFE. To investigate the effect of CITED or control formulations on BMDCs, CellTrace™ CSFE labeled BMDCs were pulsed with 1 μg/mL lipopolysaccharide (LPS) and 40 μg/mL ovalbumin (OVA) overnight. Activated BMDCs were then co-cultured with MDSCs at a MDSC-to-DC ratio of 1:1 for 24 hours. The expression of CD80 and CD40 on CellTrace™ CSFE labeled BMDCs were quantified by antibody staining against CD11c (PerCP, category # 117326, clone # N418, BioLegend), CD40 (BV421, category # 16-10A1, clone # 16-10A1, BioLegend), and CD80 (BV650, category # 124641, clone # 3/23, BioLegend) followed by flow cytometry analysis (Aurora, Cytek).

RNA-seq data analysis. Raw sequencing reads were processed using Salmon (v1.4.0) for pseudoalignment and transcript quantification. To enhance mapping accuracy and reduce spurious alignments, decoys were introduced during transcriptome index generation. Decoy sequences, including the genome assembly and other non-transcribed regions, were added to the index using the --decoys option in Salmon. The transcriptome reference and decoy sequences were obtained from GENCODE (Genome sequence, primary assembly (GRCm39)). Quantified transcript-level abundances were imported into R (v4.3.2) using the tximport package and aggregated to gene-level counts based on the GENCODE annotation. Quality control was performed to ensure the robustness of the data. Genes with low expression across samples were filtered out using a predefined threshold (genes with fewer than 2 counts across all samples were excluded). Normalization of the count data was conducted using the variance stabilizing transformation (VST) function from the DESeq2 package (v1.42.1), which log-transforms the data and stabilizes variance across different levels of gene expression. Differential expression analysis (DEG) was performed using the DESeq2 package (v1.42.1). Gene-level count data were modeled using a negative binomial distribution, and hypothesis testing was conducted using the Wald test. Genes with an adjusted p-value (Benjamini-Hochberg correction) below 0.05 and an absolute log2 fold change (log FC) ≥1 were considered differentially expressed. Gene Set Enrichment Analysis (GSEA) was performed on a ranked gene list derived from the differential expression analysis using the GSEA function from the ClusterProfiler package (v4.10.1) in R. Genes were ranked based on the Wald statistic, and only those with a base Mean > 50 were included in the analysis. This filtering ensured that the enrichment analysis focused on genes with sufficient expression levels, identifying pathways relevant to the ranked gene set. The analysis was conducted using the Biological Process (BP) ontology and genes annotated to Mus musculus (mouse) genome, with gene symbols converted to Entrez IDs.

Histology, immunohistochemistry, and quantification. Lumbar spinal cord sections from EAE mice after different treatments were harvested, fixed in 10% formalin at room temperature for 24 hours, and then processed through a graded series of ethanol, cleared in xylene, and embedded in paraffin. Paraffin-embedded tissues were sectioned at 5 μm thickness using a microtome and mounted on glass slides.

For histological analysis, sections were deparaffinized in xylene, rehydrated through a graded ethanol series, and stained with Hematoxylin and Eosin (H&E) following standard protocols. Additionally, Luxol fast blue staining was performed to assess myelin content. For Luxol fast blue staining, tissue samples were processed in the ASP-300 automates tissue processor (Leica Biosystems) using a preset standard protocol and embedded into paraffin blocks. The tissue was sectioned at 5 μm on positively charged slides, dried and baked at 60°C for an hour. A set of sections was deparaffinized and rehydrated to 95% ethanol using a preset protocol on the Leica Autostainer XL automated stainer (Leica Biosystems). Slides were incubated in Luxol Fast Blue (Newcomer Supply Inc, #12218) for two hours at 60°C, rinsed in 95% ethanol and distilled water and consecutively differentiated in 0.05% working solution on Lithium Carbonate (Newcomer Supply Inc, #12215) and 70% ethanol

until gray and white matter can be clearly distinguished. Slides were then counterstained with Nuclear Fast Red (Newcomer Supply Inc, #1255) for 5 minutes, washed, dehydrated on the Autostainer XL, and mounted with Micromount (Leica Microsystems, #3801730).

For immunohistochemistry (IHC) analysis, adjacent slides were used for immunohistochemistry with anti-Myelin Basic Protein antibody (1:1000, Cell Signaling, #78896). Staining was performed with a BOND Polymer Refine Detection Kit (Leica, #DS9800) on a BOND RX automated stainer (Leica Biosystems) according to the preset protocol. After deparaffinization, sections were subjected to heat-based antigen retrieval with BOND Epitope retrieval buffer 1 (pH 6.0, Leica Biosystems, # AR9961) for 20 min at 99°C. Endogenous peroxidase activity and non-specific binding was blocked by sequentially treating samples with peroxidase block (BOND Polymer Refine Detection Kit) and protein block (Background Sniper, Biocare Medical, #BS966) for 15 min at room temperature. Sections were then incubated with the primary antibody for 30 min. After several washes, the signal detection was performed with anti-rabbit-Poly-HRP and DAB from the BOND Polymer Refine Kit by incubating sections for 15 min and 10 min at room temperature correspondingly, and counterstained with hematoxylin. All slides were dehydrated on Autostainer XL and mounted with Micromount media. Stained sections were scanned using Leica Aperio AT2. Images were analyzed using Indica Labs HALO image analysis platform to quantify cell counts and staining intensity.

Supplementary Figures

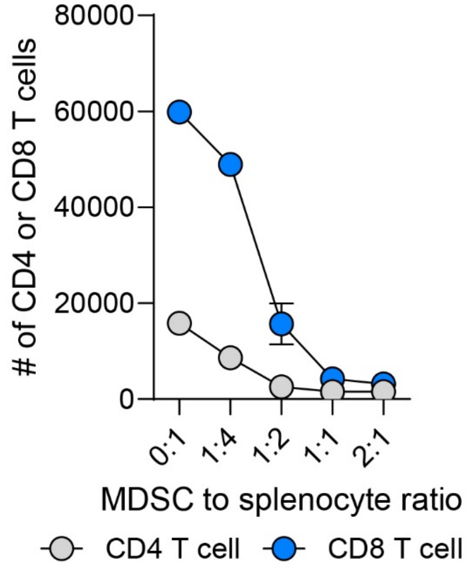

Fig. S1. MDSC-like cells inhibit T cell proliferation. The number of CD4 and CD8 T cells after co-culture with MDSCs at different MDSC to splenocyte ratios for 5 days was shown. Data are presented as mean ± SEM.

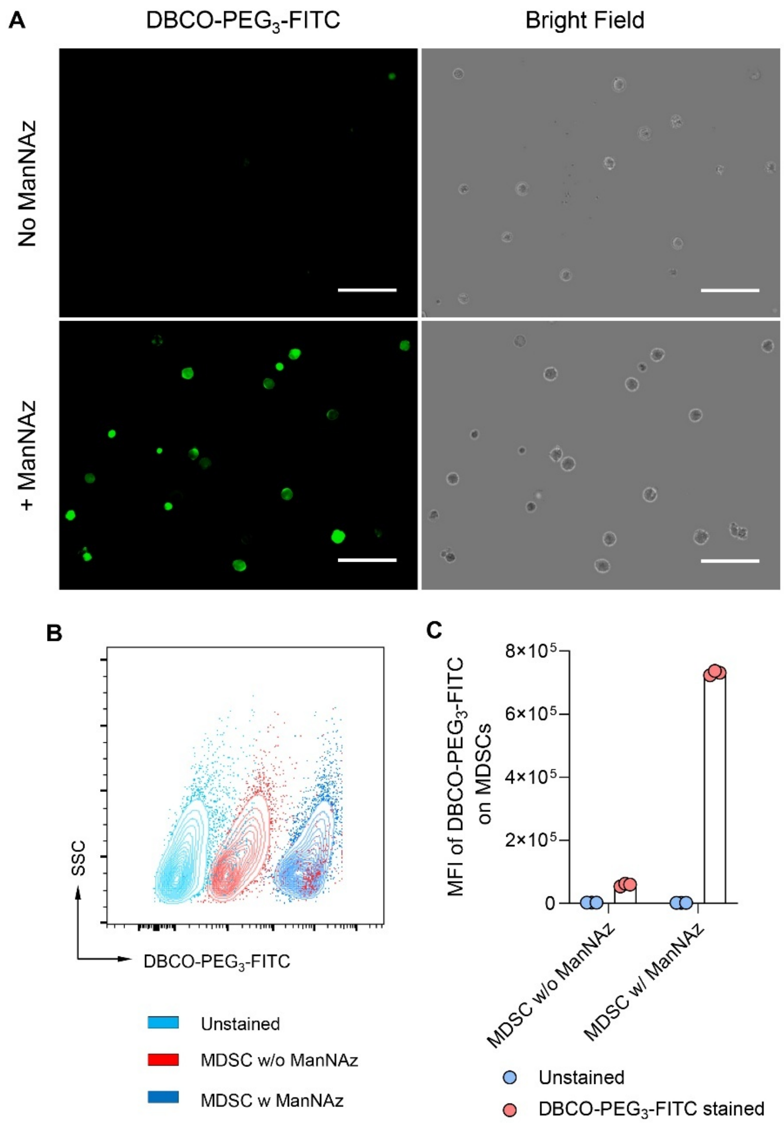

Fig. S2. Reactive azido functional groups were displayed on MDSCs following incubation with Ac_4ManNAz. A, Fluorescence microscopic images of untreated or Ac_4ManNAz-treated MDSCs after staining with DBCO-PEG_3-FITC. Scale bar: 50 µm. **B**, Representative flow cytometry plot of MDSCs stained with DBCO-PEG_3-FITC. **C**, Quantification of the relative number of reactive azido groups (indicated by the mean fluorescence intensity of DBCO-PEG_3-FITC) on untreated or Ac_4ManNAz-treated MDSCs. Data in (**C**) are presented as mean ± SEM.

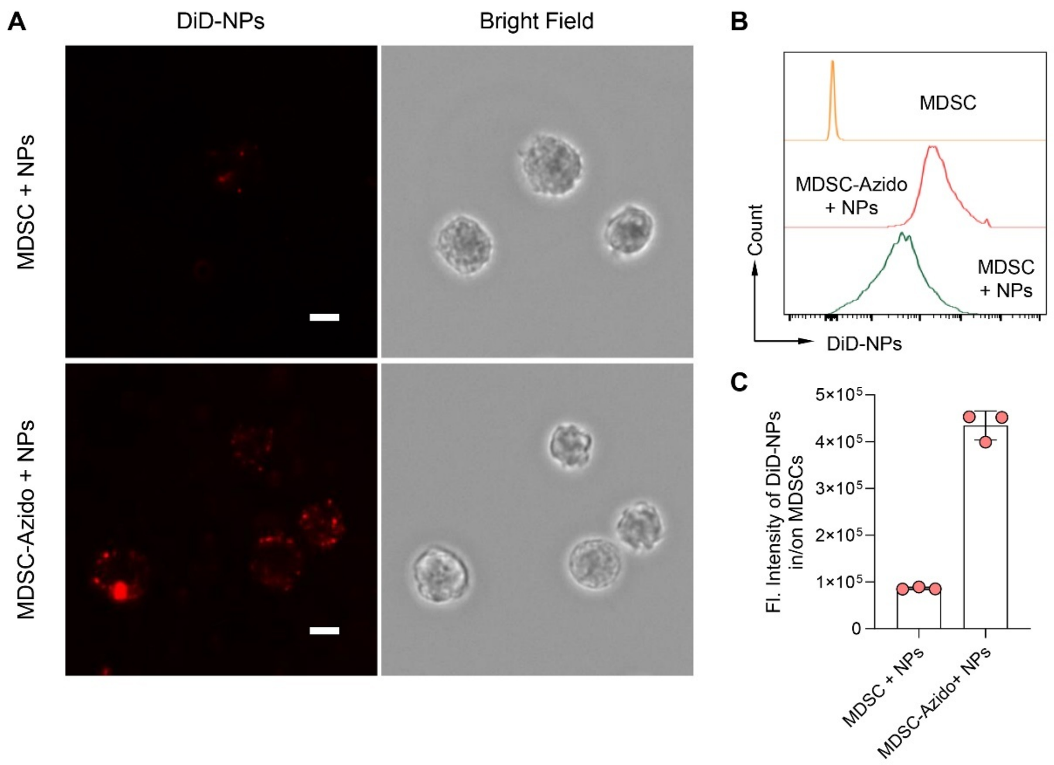

Fig. S3. MDSCs show limited phagocytosis of the immunomodulatory NPs and the azido-DBCO click chemistry method enhances NP loading onto MDSCs. **A**, Fluorescence microscopic images of MDSCs or MDSC-Azido after incubation with NPs for 1 hour. Scale bars: 5 µm. **B**, Representative flow cytometry plots showing the relative number of NPs associated with MDSCs or MDSC-Azido after 1 hour of incubation. **C**, Quantification of the relative number of NPs (indicated by the mean fluorescence intensity of DiD) on untreated or azido-functionalized MDSCs. Data in (**C**) are presented as mean ± SEM.

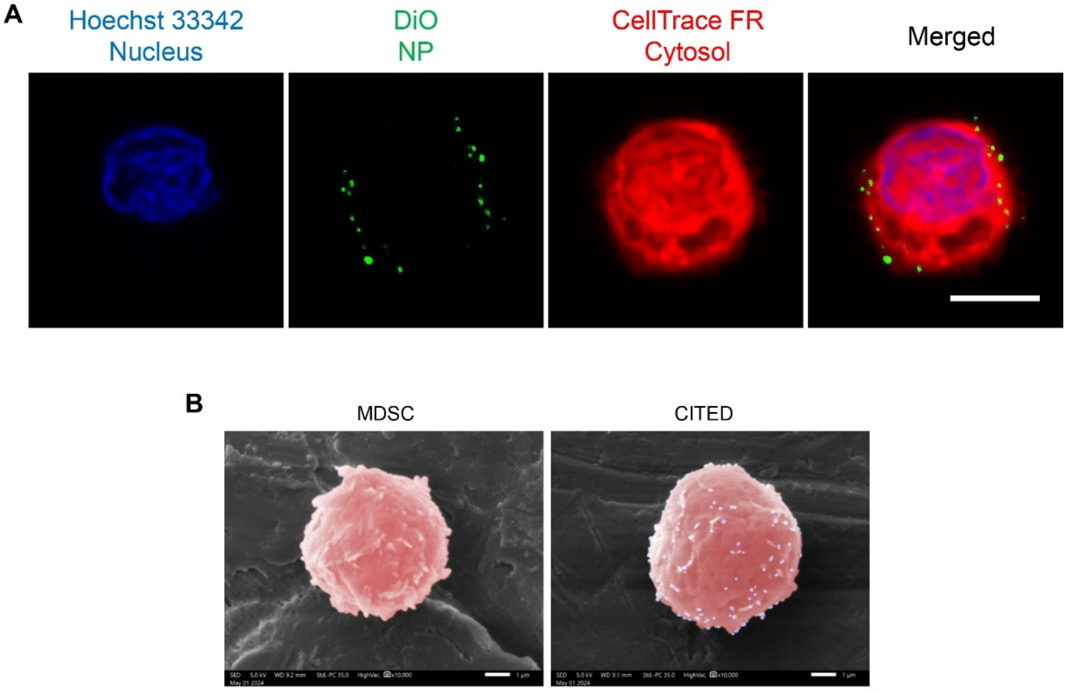

Fig. S4. Representative confocal laser scanning microscopic (CLSM) and scanning electron microscopic (SEM) images of M-MDSC carrying immunomodulatory NPs on their surface. A, CLSM image of an M-MDSC carrying NPs. Scale bar: 5 µm. **B**, Pseudo-colored SEM image of an M-MDSC carrying NPs. P-MDSC and NPs were pseudo-colored in pink and purple, respectively. Scale bars: 1 µm.

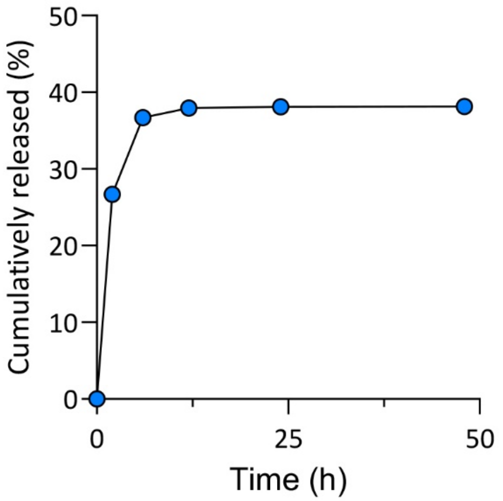

Fig. S5. Cumulative release of rapamycin from CITED in PBS containing 10% FBS. Data are presented as mean ± SEM. n=3 independent samples.

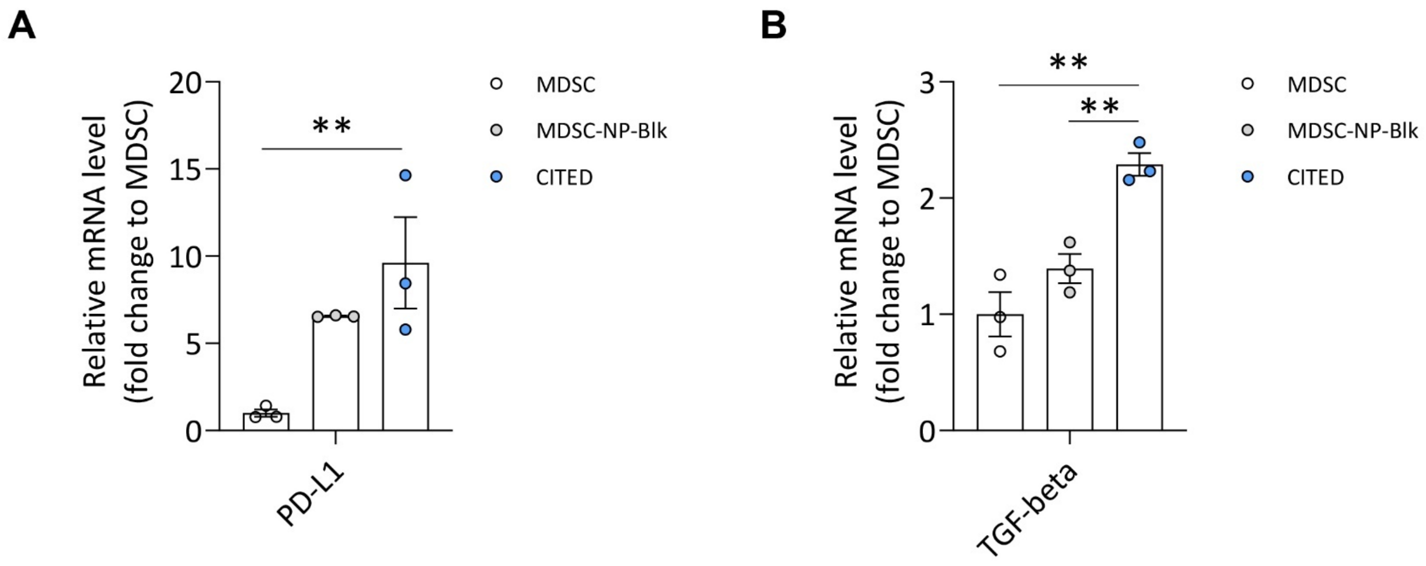

Fig. S6. Relative mRNA level of PD-L1 and TGF-β in different MDSC formulations. mRNA level of PD-L1 (**A**) and TGF-β (**B**) in MDSCs 24 hours after NP conjugation was quantified by RT-qPCR. Significantly different (One-way ANOVA with Dunnett test): ** $p < 0.01$. Data in (**A, B**) are presented as mean ± SEM.

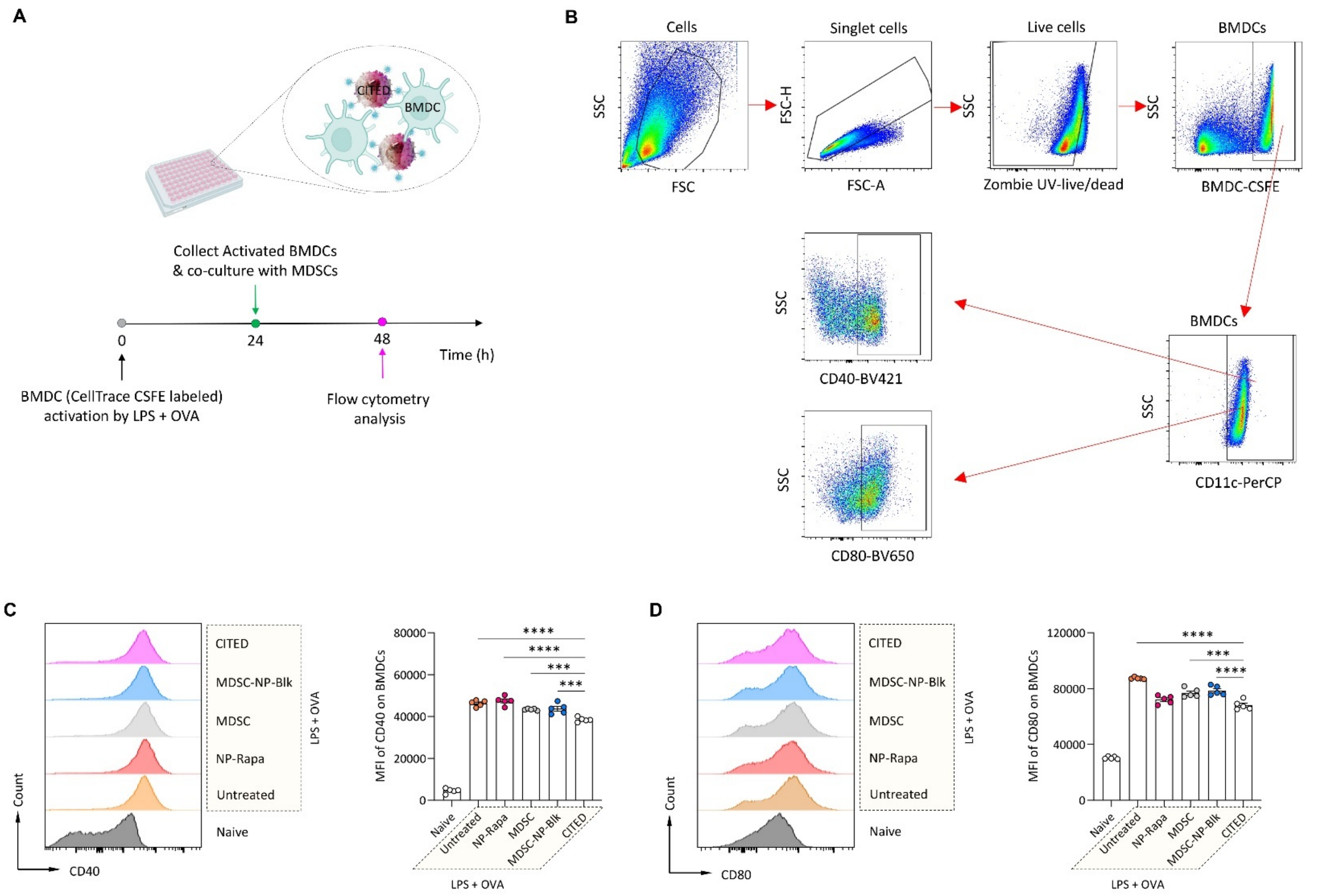

Fig. S7. CITED led to downregulated expression of co-stimulatory molecules on DCs when co-cultured with activated BMDCs. **A**, Schematic showing the experimental design. Created in BioRender. Zhao, Z. (2025) https://BioRender.com/2tie67h. **B**, Flow cytometry gating strategy to identify DCs and analyze CD40/CD80 expression on their surface. **C**, Representative flow cytometry histograms showing the expression of CD40 on BMDCs treated with different formulations. Mean fluorescence intensity (MFI) of CD40 is quantified and shown. **D**, Representative flow cytometry histograms showing the expression of CD80 on BMDCs treated with different formulations. MFI of CD80 is quantified and shown. Data in (**C, D**) are presented as mean ± SEM. For (**C-D**), significantly different (one-way ANOVA with Dunnett test): *** $p < 0.001$, **** $p < 0.0001$.

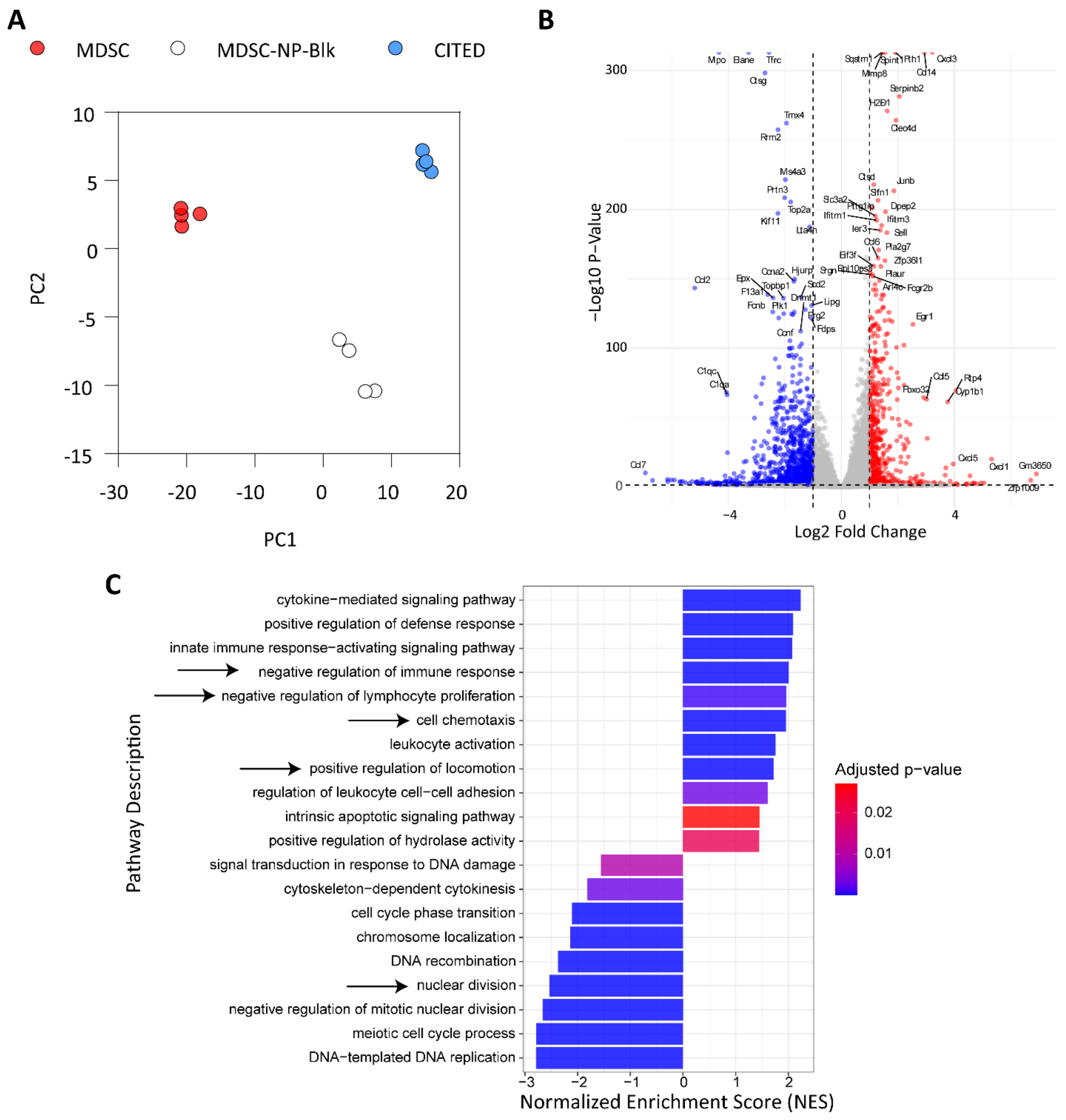

Fig. S8. Rapa NP conjugation altered the transcriptome of MDSCs. A, Principal component analysis (PCA) plot showing the distinct clustering of biological replicate samples across three groups: MDSC, MDSC_NP_Blk, and CITED (n=4 biologically independent replicates for each group). Samples are color-coded by groups to highlight the clustering patterns. **B**, Differential expression analysis comparing CITED and MDSC groups. Genes with an adjusted p-value (adj. $p \leq 0.05$) and absolute log2 fold change ($|LogFC| > 1$) were classified as differentially expressed. Red points indicate genes upregulated in the CITED group and blue points indicate genes upregulated in the MDSC group. **C,** Bar plot showing gene set enrichment analysis (GSEA) results between the CITED and MDSC groups. Positive normalized enrichment score (NES) values reflect pathways upregulated in the CITED group, while negative NES values correspond to pathways enriched in the MDSC group.

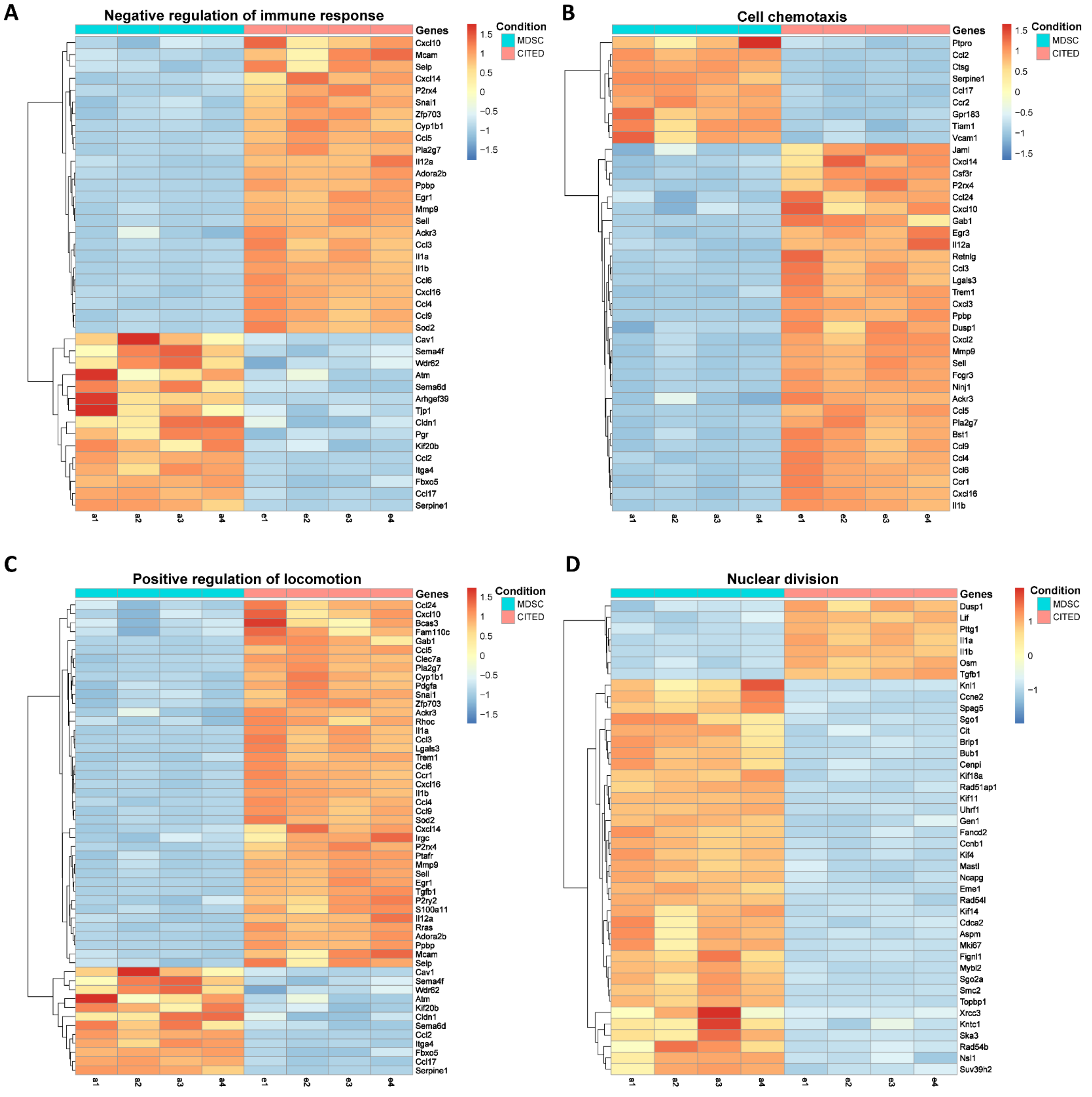

Fig. S9. Key biological pathways in MDSCs altered by Rapa NP conjugation. Heatmaps depicting the normalized expression levels (log2-transformed) of differentially expressed genes within key biological pathways, including negative regulation of immune response (**A**), cell chemotaxis (**B**), positive regulation of locomotion (**C**), and nuclear division (**D**). Each row corresponds to a gene, and columns represent biologically independent samples (n=4).

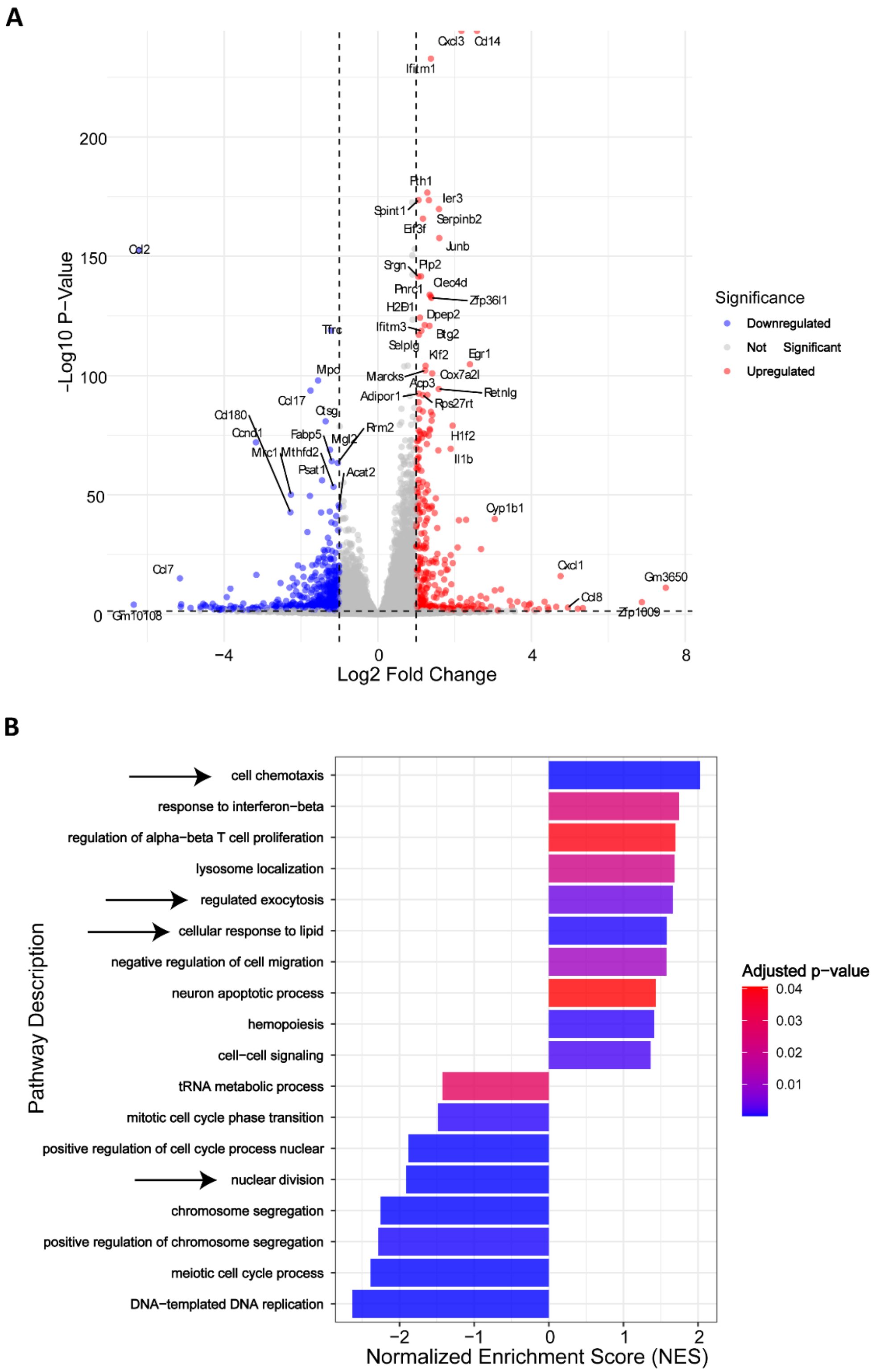

Fig. S10. Conjugation of blank NPs to MDSC altered their transcriptome. A, Differential expression analysis comparing MDSC_NP_Blk and MDSC groups. Genes with an adjusted p-value (adj. p $\leq$0.05) and absolute log2 fold change (|LogFC|>1) were classified as significantly differentially expressed. Red points represent genes upregulated in the MDSC_NP_Blk group, while blue points indicate genes upregulated in the MDSC group. **B,** Bar plot showing GSEA results between the MDSC_NP_Blk and MDSC groups. The normalized enrichment scores (NES) are used to indicate pathway activity between groups. Positive NES values reflect pathways upregulated in the MDSC_NP_Blk group, while negative NES values correspond to pathways upregulated in the MDSC group.

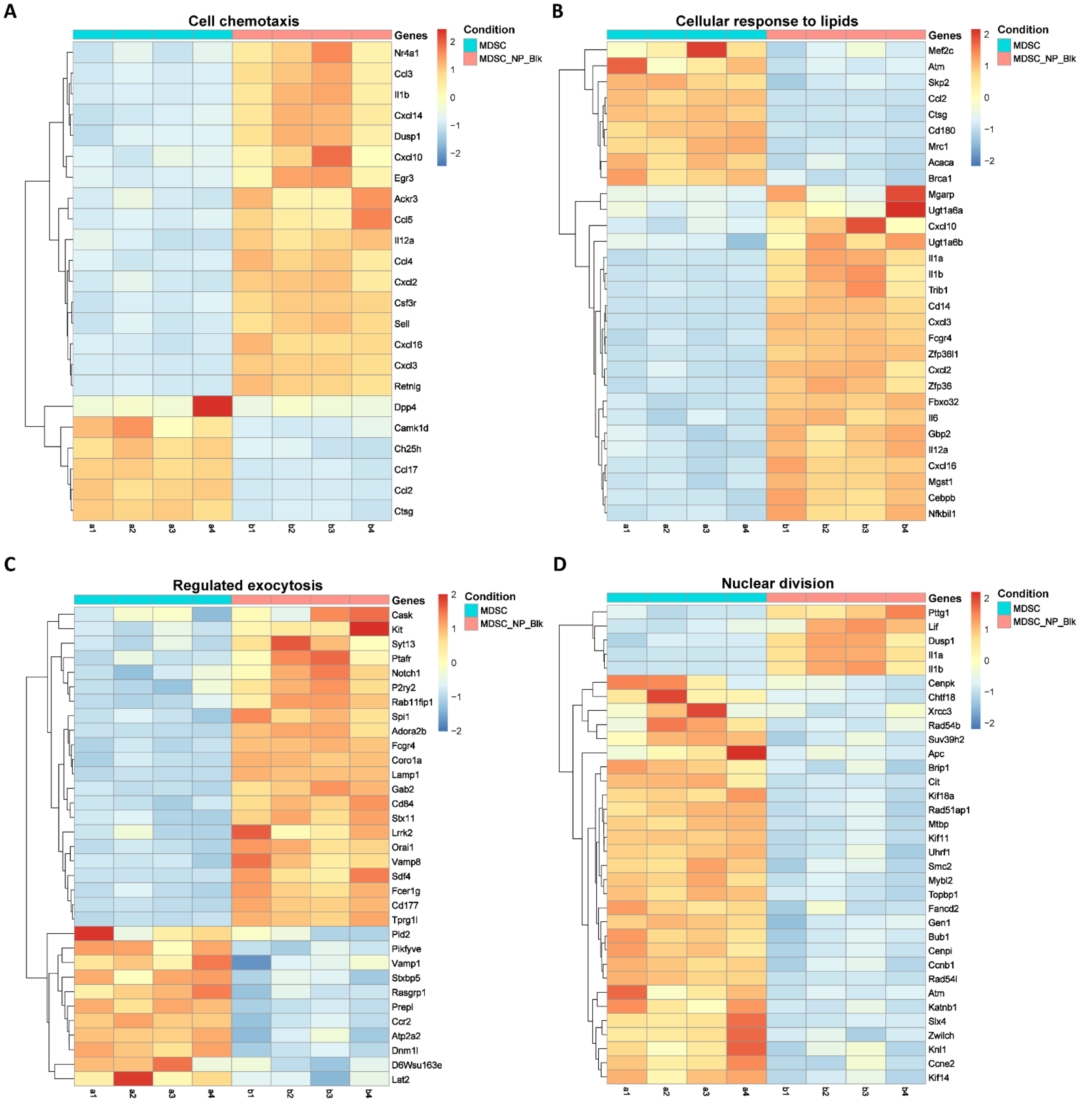

Fig. S11. Key biological pathways in MDSCs altered by blank PLGA NP conjugation. Heatmaps showing the normalized expression levels (log2-transformed) of differentially expressed genes within key biological pathways, including cell chemotaxis (**A**), cellular response to lipids (**B**), regulated exocytosis (**C**), and nuclear division (**D**). Rows represent genes, while columns correspond to individual samples.

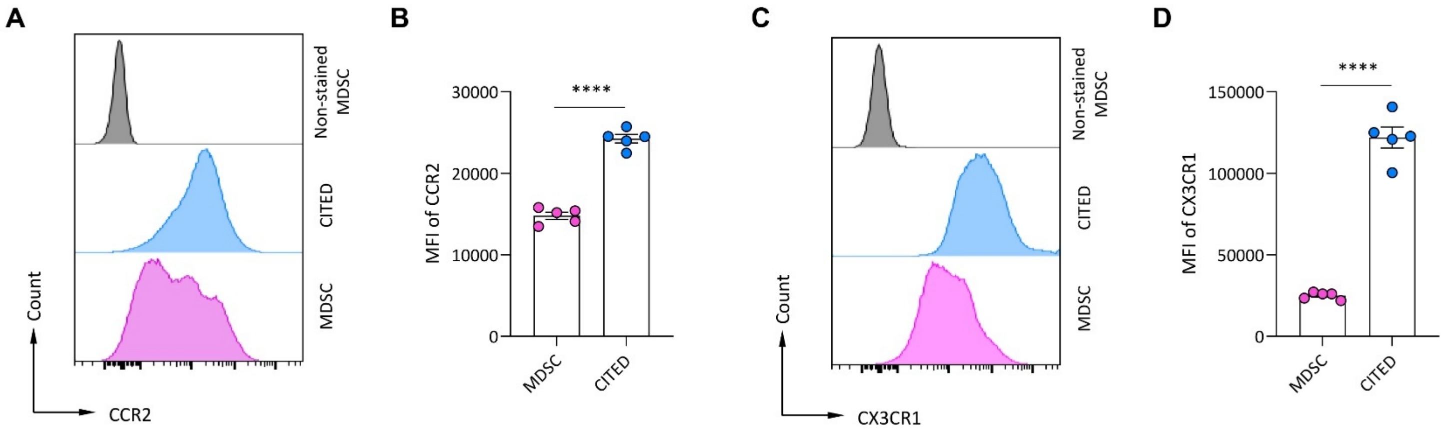

Fig. S12. Rapa NP conjugation upregulated the expression of CCR2 and CX3CR1 on MDSCs. A, Representative flow cytometry histogram showing the expression of CCR2. **B**, MFI of CCR2 on MDSCs. **C**, Representative flow cytometry histogram showing the expression of CX3CR1. **D**, MFI of CX3CR1 on MDSCs. Data in (**B, D**) are presented as mean ± SEM. For (**B, D**), significantly different (two-tailed student's t test): **** $p < 0.0001$.

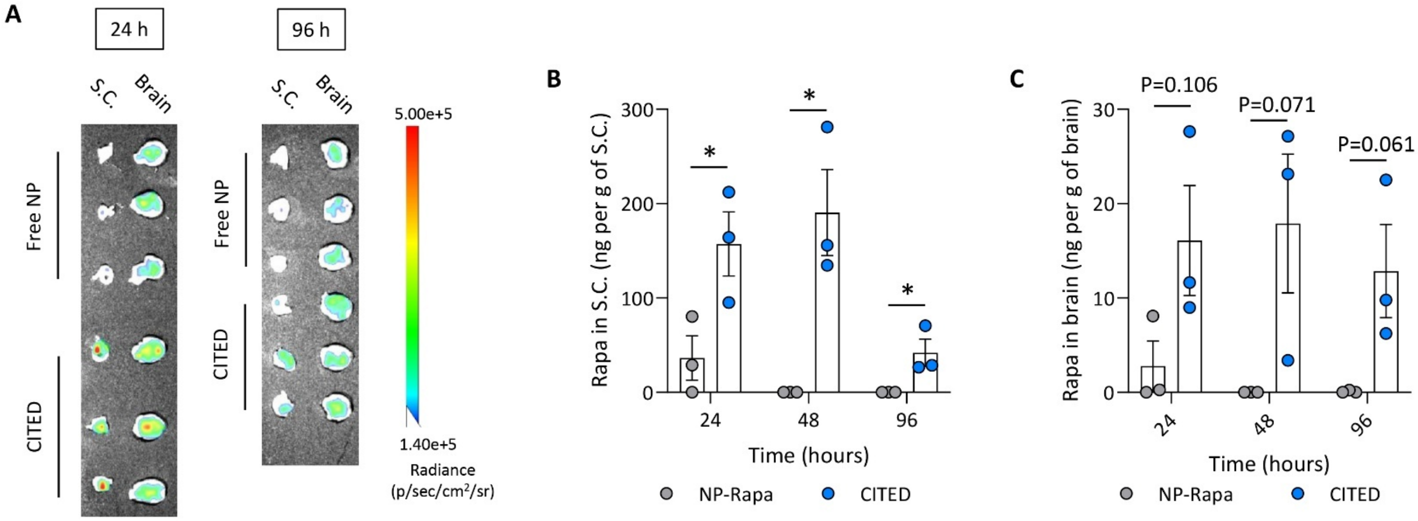

Fig. S13. CITED enhanced the delivery of NP-Rapa to the inflamed CNS in EAE mice. **A,** Lago-X images showing the accumulation of DiR-labeled NP-Rapa in the brain and spinal cord (S.C.) 24 or 96 hours after intravenous injection. **B,** The amount of rapamycin in the spinal cord. **C,** The amount of rapamycin in the brain. For (**A-C**), n=3 biologically independent animals. Data in (**B, C**) are presented as mean ± SEM. For (**B, C**), significantly different (two-tailed student's t test): * $p < 0.05$.

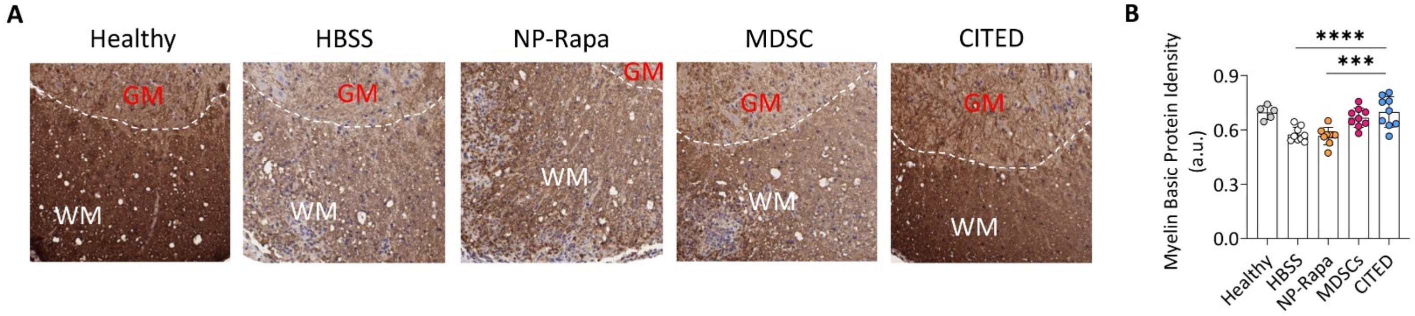

Fig. S14. CITED restored the expression of myelin basic protein (MBP) in the spinal cord of EAE mice. A, Representative immunohistology MBP staining images showing the expression of MBP in the spinal cord. WM: white matter; GM: gray matter. **B,** Quantification of MBP intensity. Data in (**B**) are presented as mean ± SEM. For (**B**), significantly different (one-way ANOVA with Dunnett test): *** $p < 0.001$, **** $p < 0.0001$. For (**B**), n=5 biologically independent animals for the Healthy group; n=9 biologically independent animals for the other groups.

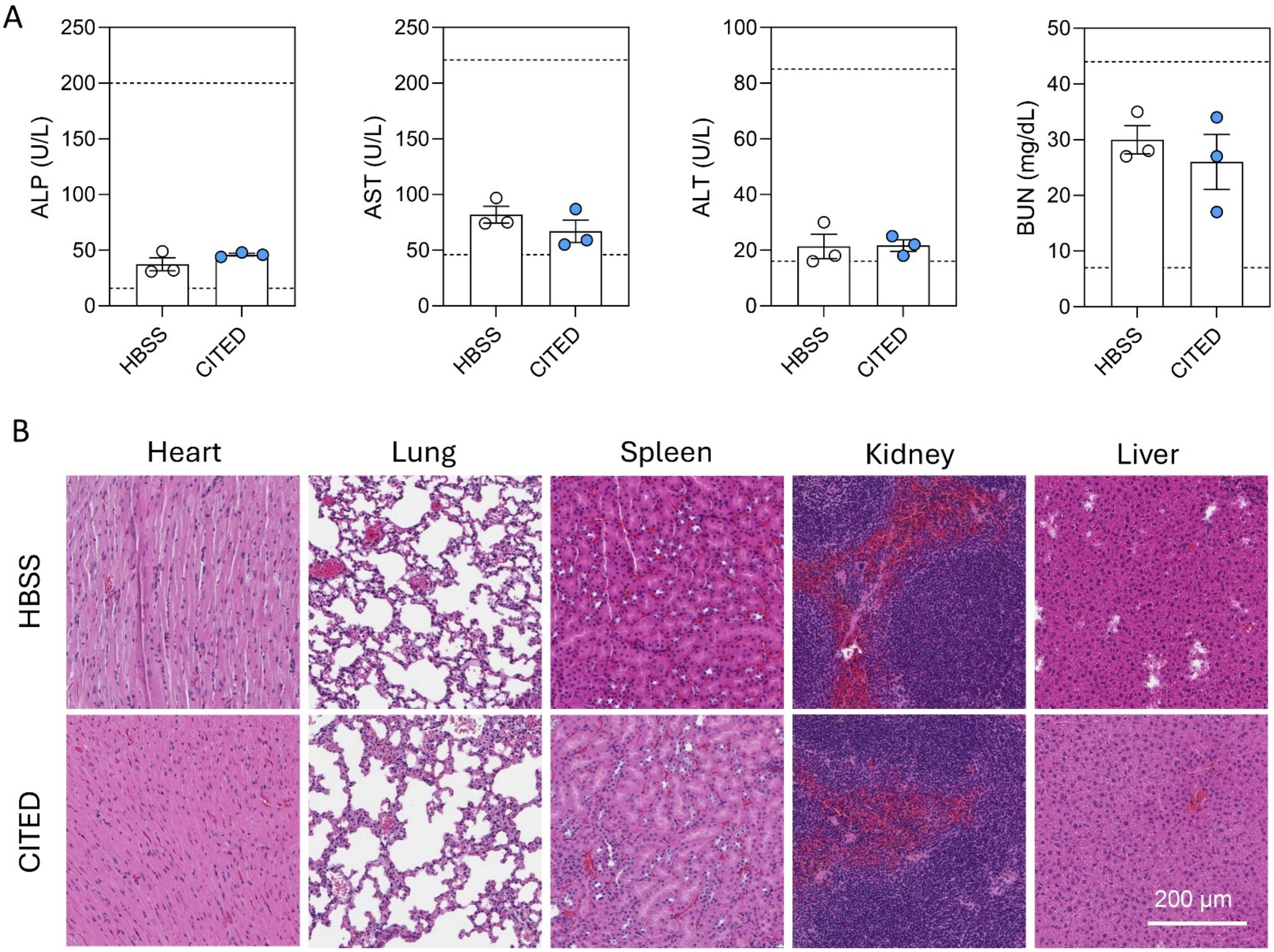

Fig. S15. Evaluation of the safety of CITED. A, Hepatic and renal functional evaluations of mice treated by CITED via blood chemistry analysis. Dashed lines indicate normal range. ALP: alkaline phosphatase; AST: aspartate aminotransferase; ALT: alanine transaminase; BUN: blood urea nitrogen. Mice were treated according to the same schedule shown in Fig. 4A and blood was collected on day 20. n=3 biologically independent animals per group. Data is presented as mean ± SEM. No statistically significant difference was detected between the HBSS and CITED groups for all four parameters (two-sided student's t test). **B,** Representative H&E staining images of major organs of EAE mice treated by CITED. Mice were treated according to the same schedule shown in Fig. 4A and organs were collected on day 25.

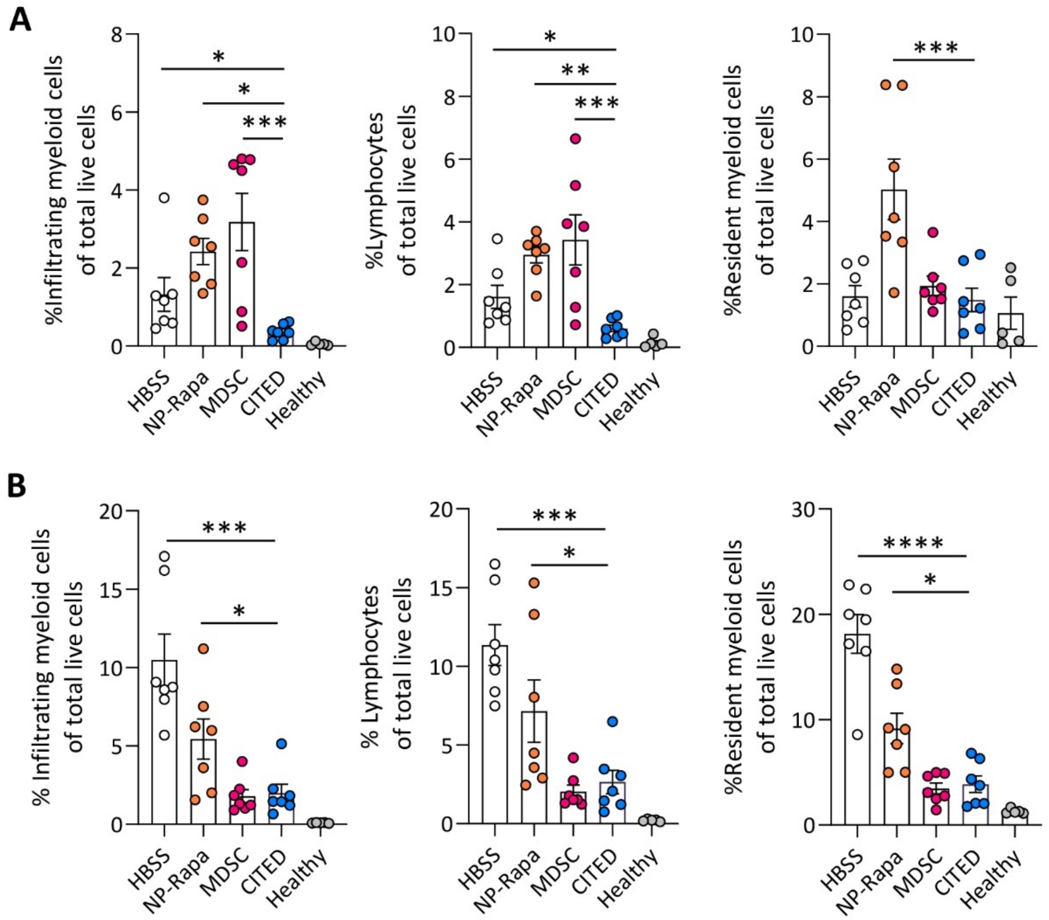

Fig. S16. Relative number of infiltrating myeloid cells, infiltrating lymphocytes, and resident myeloid cells in the spinal cord and brain of mice following different treatments. **A**, Percentage of respective cells in the spinal cord. **B**, Percentage of respective cells in the brain. Data in (**A, B**) are presented as mean ± SEM. Significantly different (one-way ANOVA with Dunnett test): * $p < 0.05$, ** $p < 0.01$, *** $p < 0.001$, **** $p < 0.0001$. The same data in the form of heatmap is shown in **Fig. 5C**. For (**A-B**), n=5 biologically independent animals for the Healthy group; n=7 biologically independent animals for the other groups.

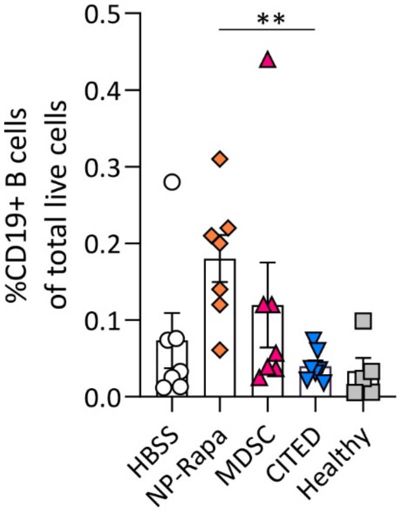

Fig. S17. Number of B cells in the spinal cord of EAE mice following different treatments. The percentage of CD19+ B cells in the mouse spinal cord was shown. Data are presented as mean ± SEM. Significantly different (one-way ANOVA with Dunnett test): ** $p < 0.01$. n=5 biologically independent animals for the Healthy group; n=7 biologically independent animals for the other groups.

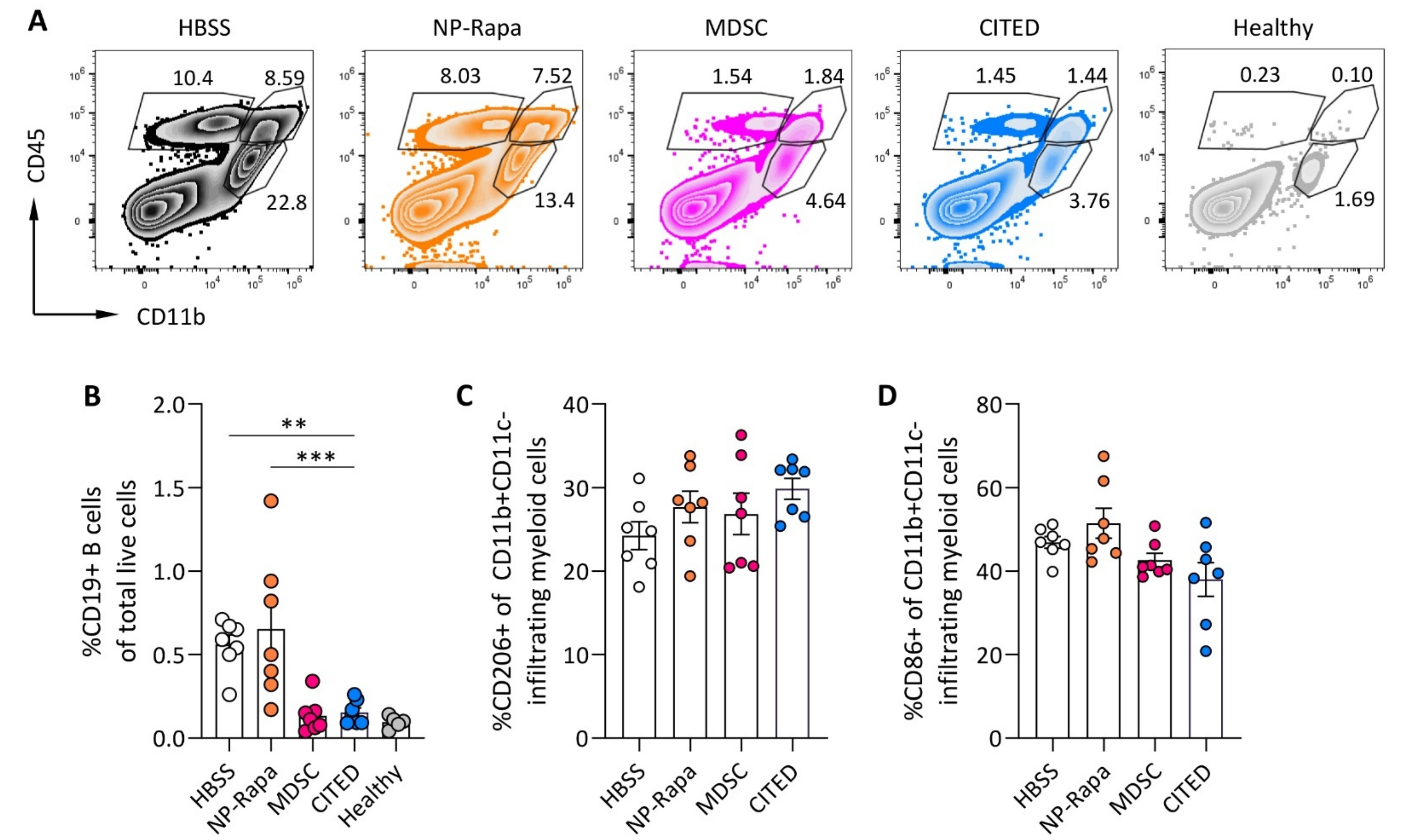

Fig. S18. Immune cell analysis in the brain of EAE mice treated by different therapies. A, Representative flow cytometry plot of infiltrating myeloid cells**,** lymphocytes, and resident myeloid cells in the brain. **B,** Relative number of CD19+ B cells in the brain. **C-D,** The phenotype of infiltrating myeloid cells in the brain. Quantitative analysis of CD206+ (M2-like) (**C**) and CD86+ (M1-like) (**D**) infiltrating CD11b+CD11c- myeloid cells in the brain. Data in (**B-D**) are presented as mean ± SEM. For (**B**), significantly different (one-way ANOVA with Dunnett test): ** $p < 0.01$, *** $p < 0.001$. For (**B-D**), n=5 biologically independent animals for the Healthy group; n=7 biologically independent animals for the other groups.

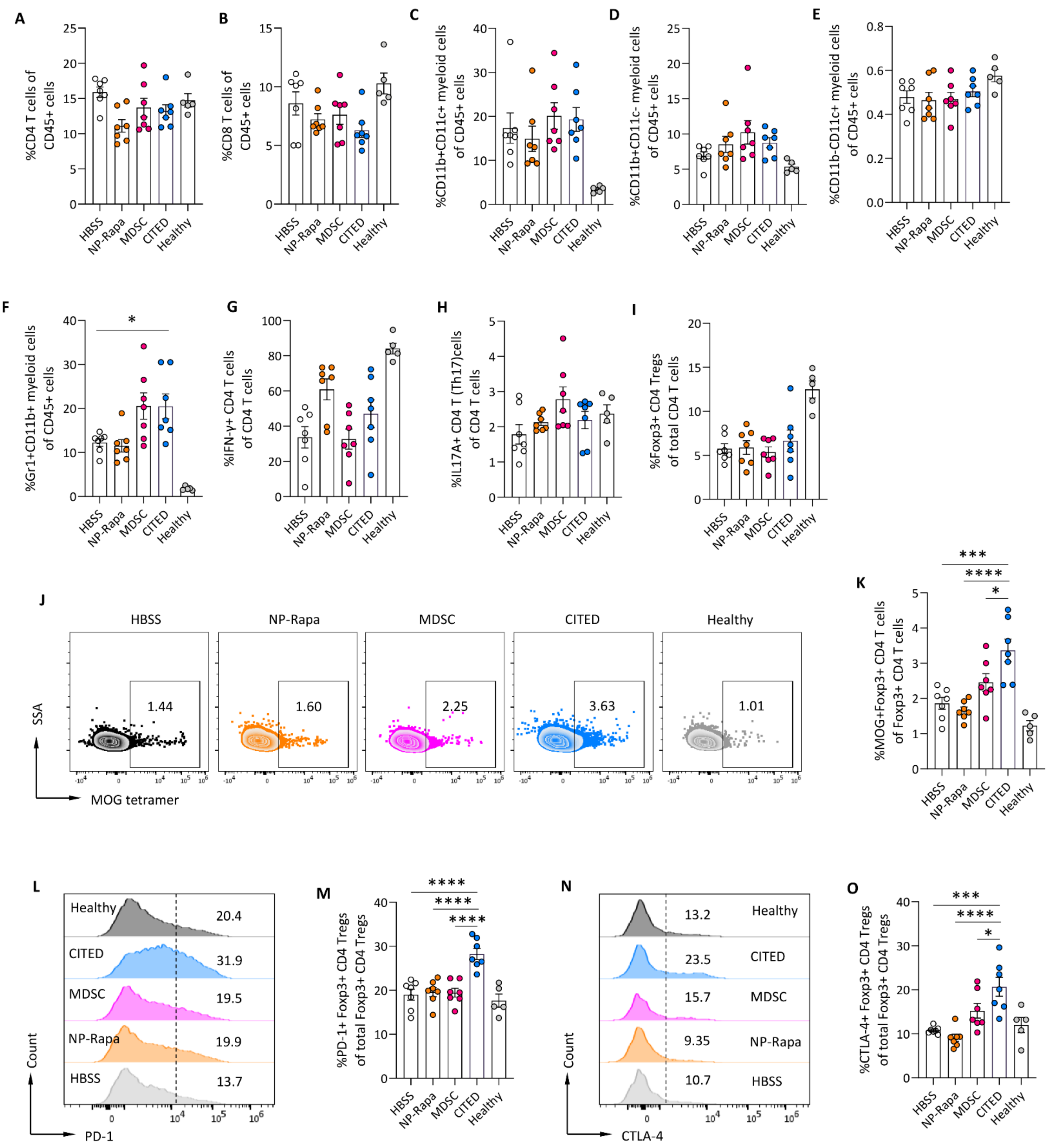

Fig. S19. Immune cell profiles in the spleen of EAE mice following different treatments. The percentage of CD4 T cells (**A**), CD8 T cells (**B**), CD11b+CD11c+ myeloid cells (**C**), CD11b+CD11c- myeloid cells (**D**), CD11b-CD11c+ myeloid cells (**E**), Gr1+CD11b+ MDSCs (**F**), IFN-γ expressing Th1 cells (**G**), Th17 cells (**H**), and Tregs (**I**) in the spleen. **J-K**, Representative flow cytometry plots (**J**) and quantification of MOG_{38-49}-specific Tregs (**K**) in the spleen. **L-O**, Representative flow cytometry plots (**L, N**) and the percentage of PD-1 or CTLA-4 positive Tregs (**M, O**) in the spleen. Data in (**A-I, K, M, O**) are presented as mean ± SEM. For (**F, K, M, O**), significantly different (one-way ANOVA with Dunnett test): * $p < 0.05$, *** $p < 0.001$, **** $p < 0.0001$. For (**A-O**), n=5 biologically independent animals for the Healthy group; n=7 biologically independent animals for the other groups.

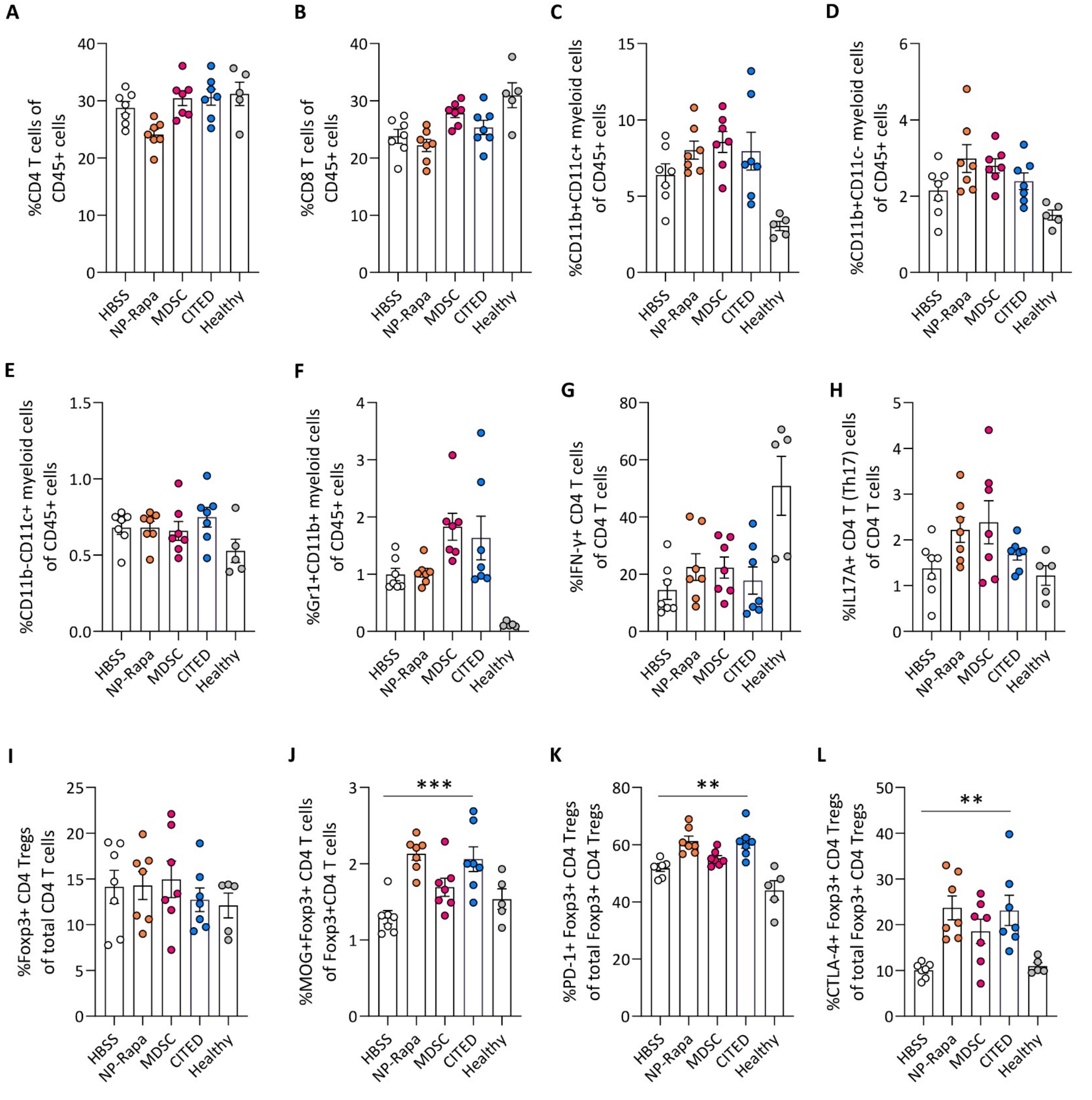

Fig. S20. Immune cell profiles in the spinal cord-draining lymph nodes (SCDLNs) of EAE mice following different treatments. The percentage of CD4 T cells (**A**), CD8 T cells (**B**), CD11b+CD11c+ myeloid cells (**C**), CD11b+CD11c- myeloid cells (**D**), CD11b-CD11c+ myeloid cells (**E**), Gr1+CD11b+ MDSCs (**F**), IFN-γ expressing CD4 T cells (**G**), Th17 cells (**H**), and Tregs (**I**) in the SCDLNs. **J,** The percentage of MOG_{38-49}-specific Tregs in the SCDLNs. **K-L,** The percentage of PD-1 or CTLA-4 positive Tregs in the CDLNs. Data in (**A-L**) are presented as mean ± SEM. For (**J-L**), significantly different (one-way ANOVA with Dunnett test): ** $p < 0.01$, *** $p < 0.001$. For (**A-L**), n=5 biologically independent animals for the Healthy group; n=7 biologically independent animals for the other groups.

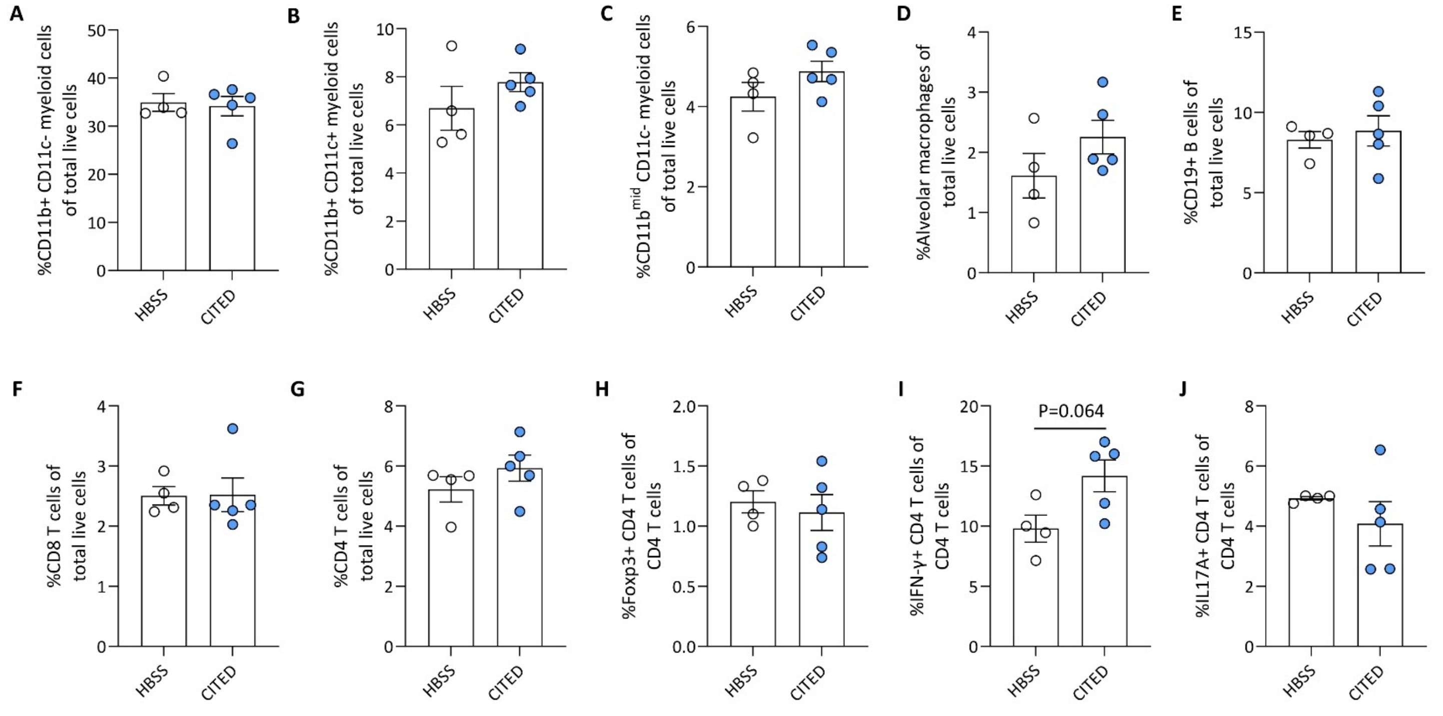

Fig. S21. Immune cell profiles in the lungs of EAE mice following HBSS or CITED treatments. The percentage of CD11b+CD11c- myeloid cells (**A**), CD11b+CD11c+ myeloid cells (**B**), CD11b^{mid}CD11c- myeloid cells (**C**), alveolar macrophages (**D**), B cells (**E**), CD8 T cells (**F**), CD4 T cells (**G**), Foxp3-expressing Tregs (**H**), IFN-γ expressing CD4 T cells (**I**), and IL-17A-expressing Th17 cells (**J**) in the lungs on day 20 post EAE induction. Mice received two doses of treatment according to the same schedule shown in Fig. 4A. Data are presented as mean ± SEM. No statistically significant difference was observed between the HBSS and CITED groups for all these immune cell types studied (two-sided student's t test). For (**A-J**), n=4 biologically independent animals for the HBSS group; n=5 biologically independent animals for the CITED group.

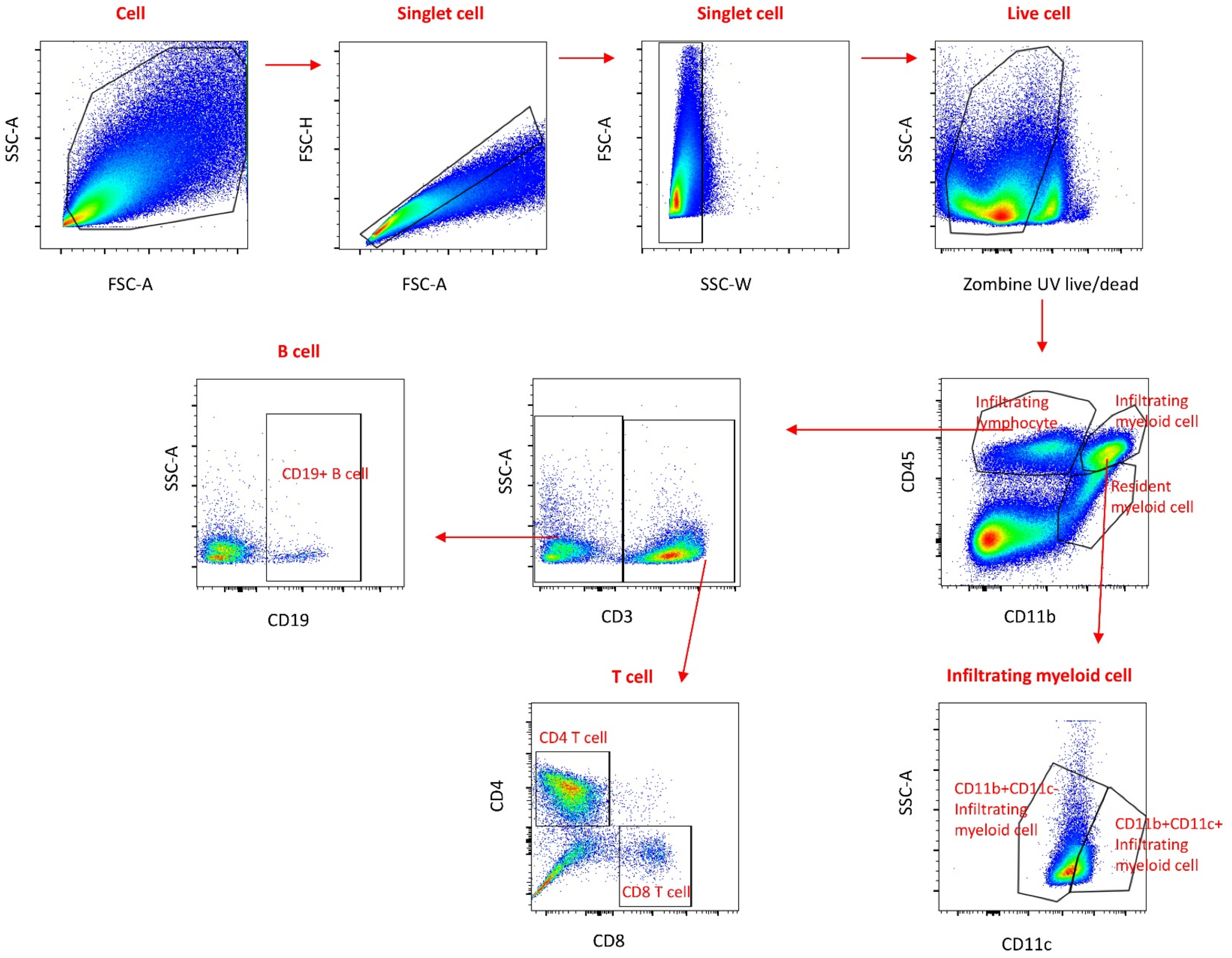

Fig. S22. Representative flow cytometry gating strategies for general immune cells in the spinal cord and brain.

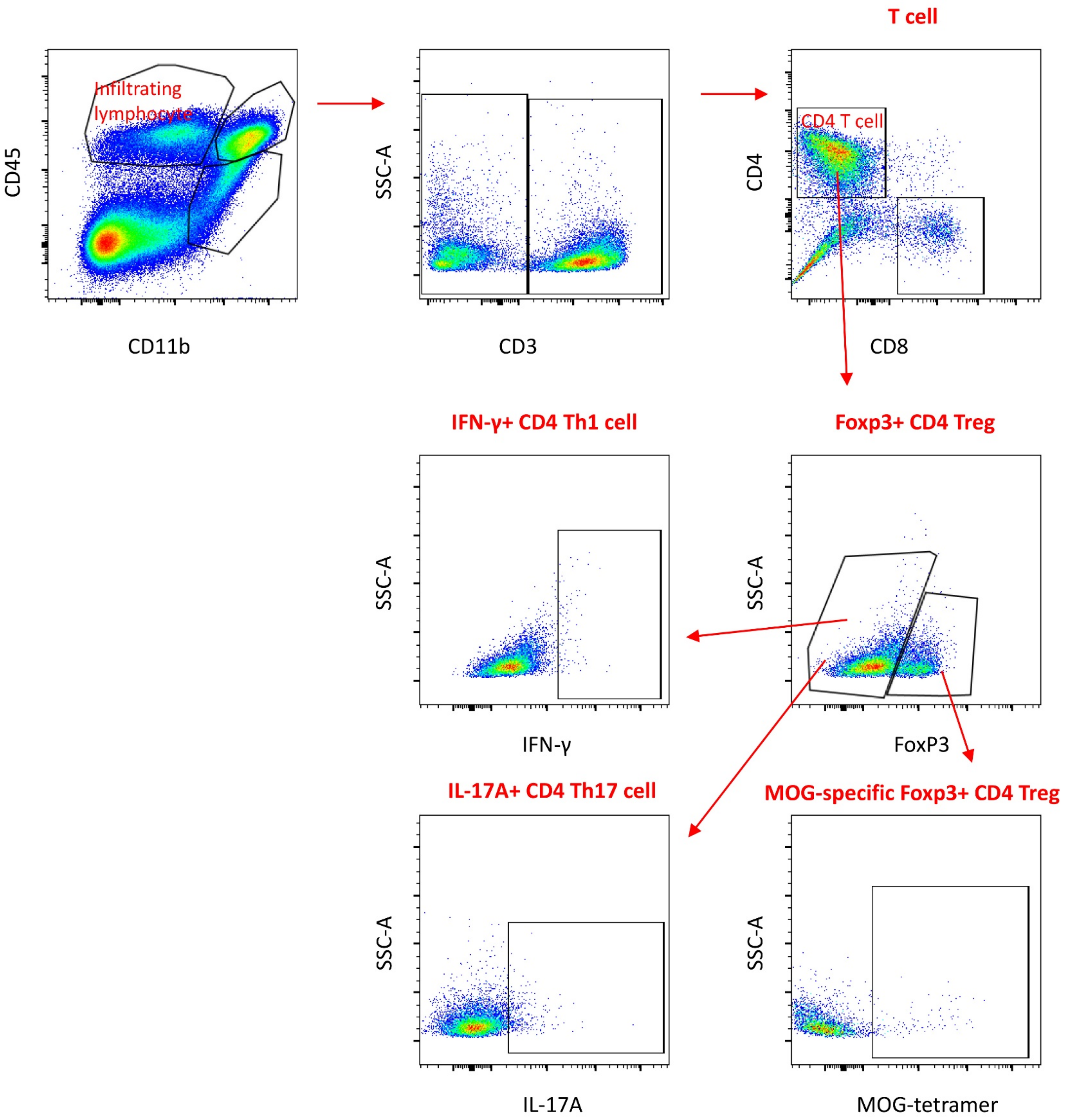

Fig. S23. Representative flow cytometry gating strategies for T cells and CD4 T cell phenotypes in the spinal cord and brain.

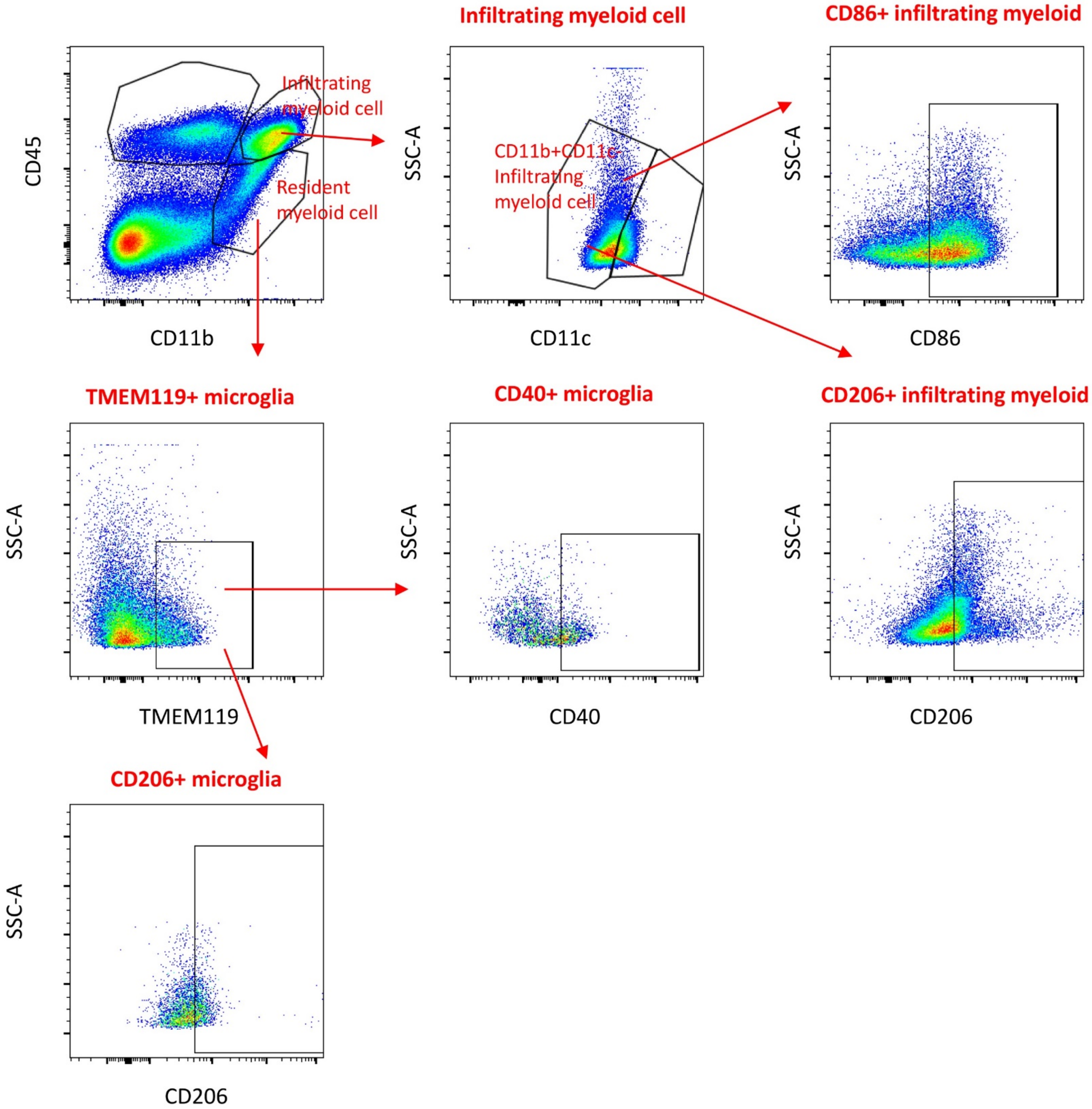

Fig. S24. Representative flow cytometry gating strategies for resident microglia and infiltrating myeloid cell phenotypes in the spinal cord and brain.

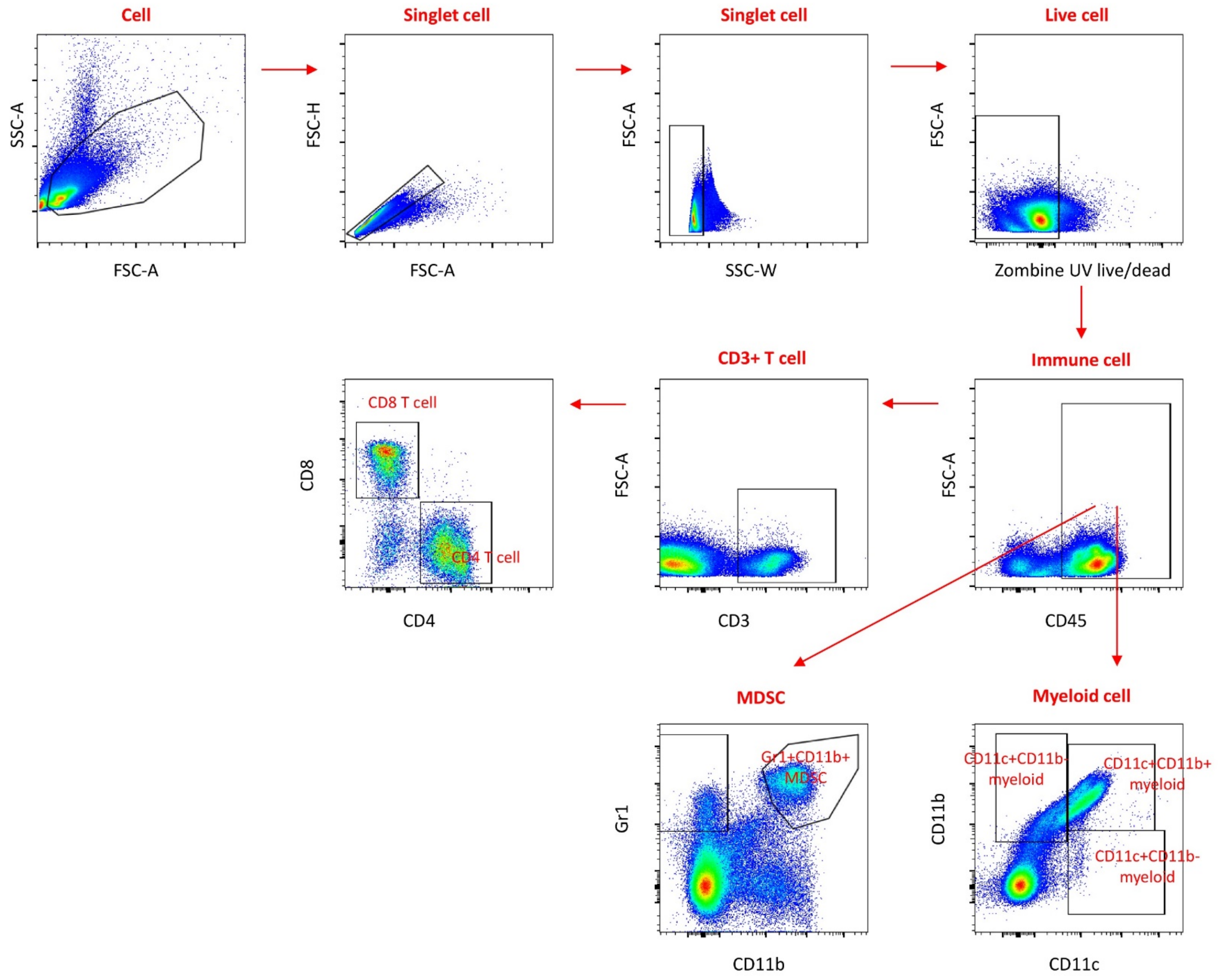

Fig. S25. Representative flow cytometry gating strategies for general immune cells in the spleen and spinal cord draining lymph nodes.

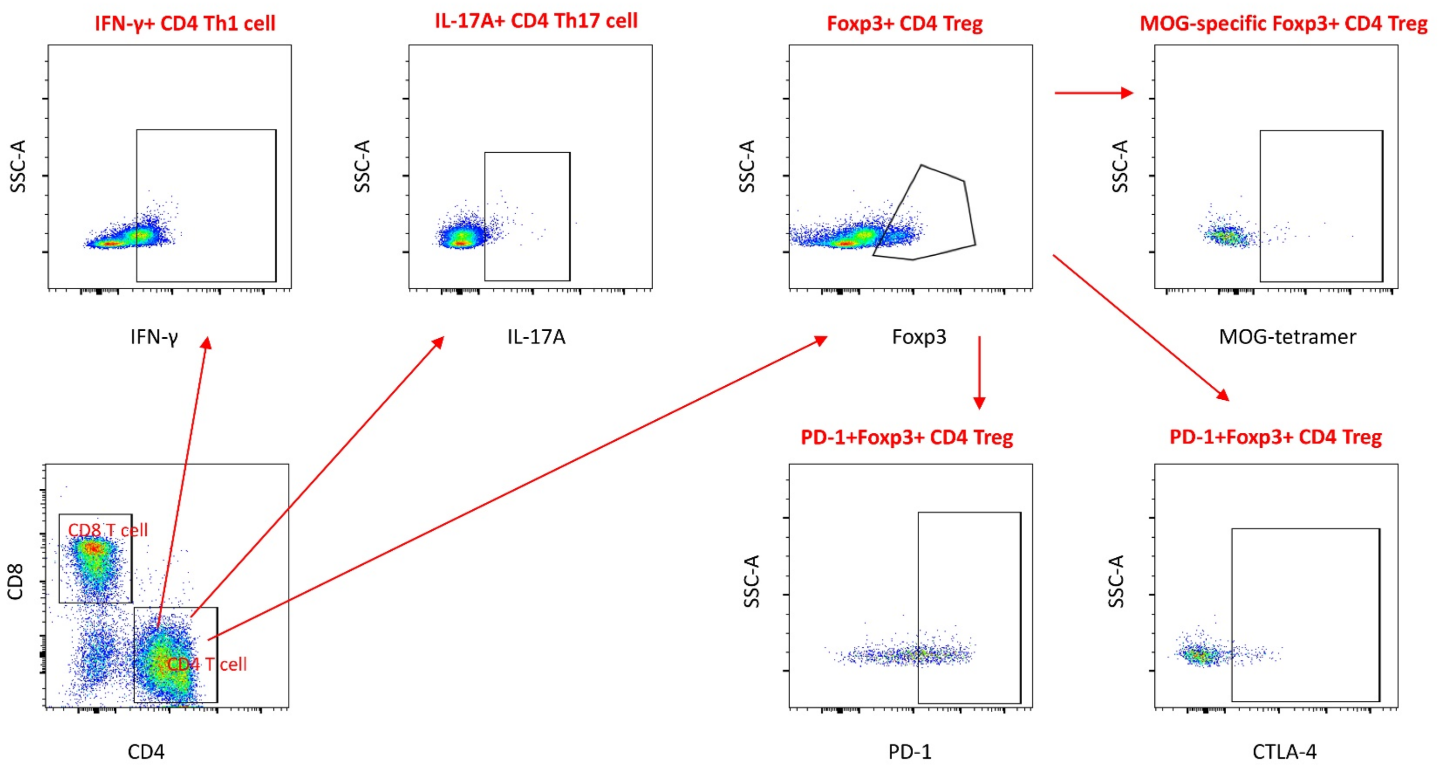

Fig. S26. Representative flow cytometry gating strategies for T cells and CD4 T cell phenotypes in the spleen and spinal cord draining lymph nodes.

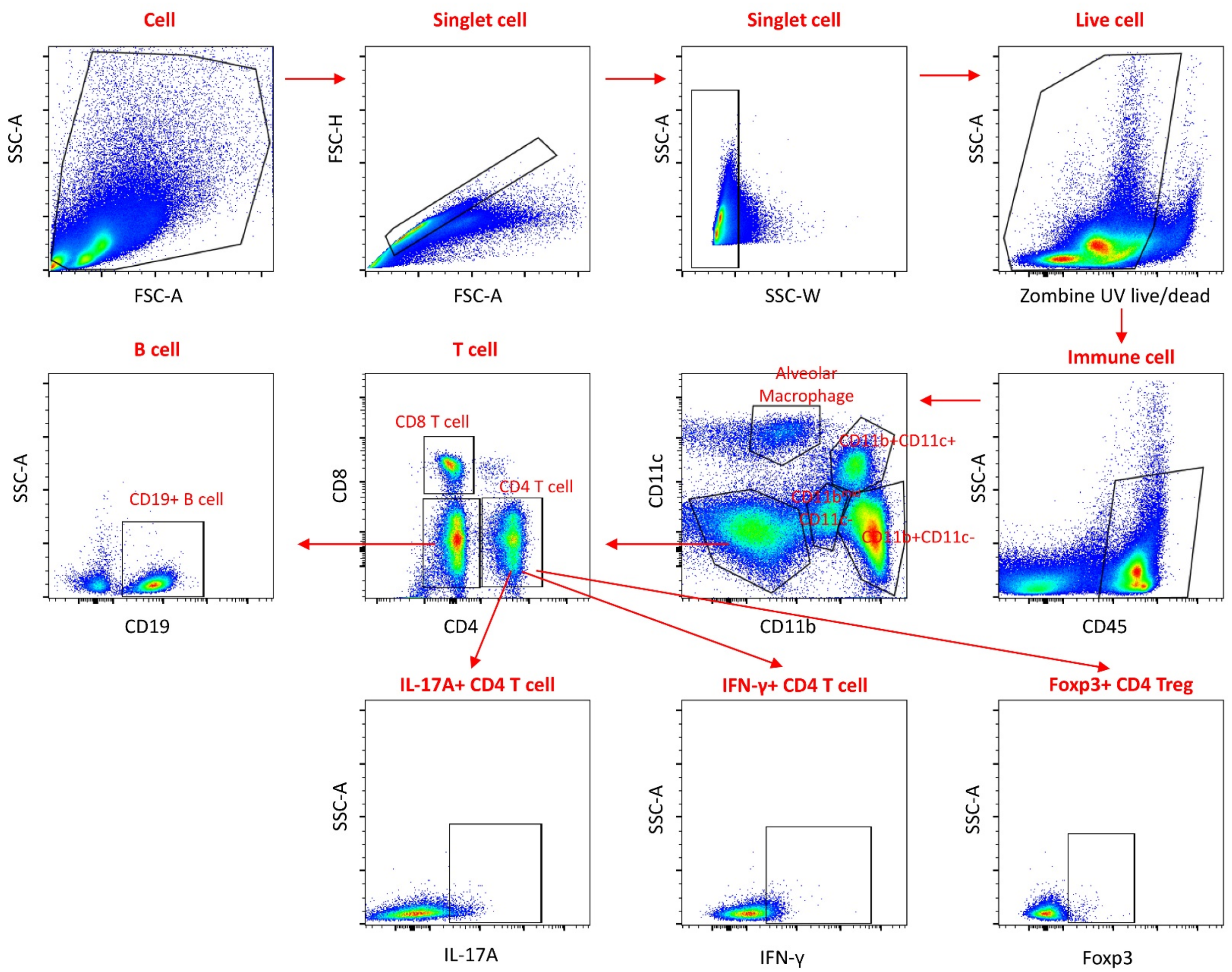

Fig. S27. Representative flow cytometry gating strategies for immune cells in the lungs.

Supplementary Table 1. Antibodies and their titrations for staining cells from mouse tissues in this work.

Fluorochrome	Antibody	Dilution Factor		Clone	Catalog number
		Spleen/Lymph node/Blood	Spinal Cord/ Brain		
Spark blue 574	anti-CD45	1:1000	1:1000	30-F11	103184 (BioLegend)
PE-Cy7	anti-TMEM119	n/a	1:20	V3RT1GOsz	25-6119-82 (eBioscience™)
PerCP-Fire 806	anti-CD11b	1:200	1:80	M1/70	101294 (BioLegend)
PerCP	anti-CD11c	1:400	1:400	N418	117326 (BioLegend)
BV510	anti-F4/80	1:80	1:200	BM8	123135 (BioLegend)
BV650	anti-CD80	1:40	1:100	16-10A1	104732 (BioLegend)
AF700	anti-CD86	1:100	1:50	GL-1	105024 (BioLegend)
BV605	anti-MHC II	1:250	1:250	M5/114.15.2	107639 (BioLegend)
BV421	anti-CD40	1:200	1:200	3/23	124641 (BioLegend)
PE-Fire700	anti-CD206	1:400	1:160	C068C2	141741 (BioLegend)
Spark blue 550	anti-CD3	1:250	1:250	17A2	100260 (BioLegend)
Spark UV 387	anti-CD4	1:400	1:1666	GK1.5	100492 (BioLegend)
Pacific Blue	anti-CD8	1:400	1:1000	53-6.7	100725 (BioLegend)
AF647	anti-Foxp3	1:50	1:50	FM-14	126408 (BioLegend)
APC-Fire810	anti-CD25	1:200	1:40	PC61	102076 (BioLegend)
BV785	anti-IL-17A	1:160	1:160	TC11-18H10.1	506928 (BioLegend)
FITC	anti-CD19	n/a	1:200	1D3/CD19	152403 (BioLegend)
APC-Fire750	anti- IFN-γ	1:400	1:400	XMG1.2	505860 (BioLegend)
BV570	anti-Gr-1	1:40	1:40	RB6-8C5	108431 (BioLegend)
PE-Cy5	anti-PD-1	1:160	1:160	29F.1A12	135256 (BioLegend)
BV711	anti-PD-L1	1:40	1:200	10F.9G2	124319 (BioLegend)
PE/Dazzle594	anti-CTLA-4	1:40	1:40	UC10-4B9	106318 (BioLegend)

Supplementary Table 2. Antibodies and their titrations for staining cell lines or primary cells in this work.

Fluorochrome	Antibody	Dilution Factor	Clone	Catalog number
FITC	anti-CD4	1:200	RM4-5	100510 (BioLegend)
APC	anti-CD8	1:160	S18018E	162306 (BioLegend)
Spark blue 550	anti-CD3	1:50	17A2	100260 (BioLegend)
Spark UV 387	anti-CD4	1:400	GK1.5	100492 (BioLegend)
AF647	anti-Foxp3	1:50	FM-14	126408 (BioLegend)
APC-Fire810	anti-CD25	1:40	PC61	102076 (BioLegend)
BV785	anti-IL-17A	1:80	TC11-18H10.1	506928 (BioLegend)
APC-Fire750	anti- IFN-γ	1:80	XMG1.2	505860 (BioLegend)
PerCP	anti-CD11c	1:40	N418	117326 (BioLegend)
BV421	anti-CD40	1:80	16-10A1	104732 (BioLegend)
BV650	anti-CD80	1:40	3/23	124641 (BioLegend)
PE/Cy7	anti-CXCR2	1:40	SA045E1	149618 (BioLegend)
PE	anti-CCR2	1:40	SA203G11	150610 (BioLegend)
BV510	anti-CD155	1:80	TX56	131527 (BioLegend)
APC	anti- FasL	1:40	MFL3	106610 (BioLegend)
BV711	anti-PD-L1	1:40	10F.9G2	124319 (BioLegend)
PE/Dazzle	anti-VISTA	1:40	MIH63	150214 (BioLegend)
Pacific blue	anti-IL-10	1:50	JES5-16E3	505020 (BioLegend)

Supplementary Table 3. Primer Sequences for qPCR Analysis.

**Genes**	**Sequence**
GAPDH	F: CCCATGTTTGTGATGGGTGTG<br>R: AGCCCTTCCACAATGCCAA
IL-1β	F: GCAACTGTTCCTGAACTCAACT<br>R: ATCTTTTGGGGTCCGTCAACT
IFN-γ	F: AAGACAATCAGGCCATCAGC<br>R: CTGGACCTGTGGGTTGTTGA
TNF-α	F: TAGCCCACGTCGTAGCAAAC<br>R: ACAAGGTACAACCCATCGGC
TGF-β	F: GGATACCAACTATTGCTTCAG<br>R: TGTCCAGGCTCCAAATATAG
PD-L1	F: CAAGTGAGAATGCTAGATGTG<br>R: TCCATCTTGAGTCTTTGGAC
